# Supplementary material for: Photochromic luminescence of organic crystals arising from subtle molecular rearrangement
Source: Nat Commun. 2024 Jun 13;15:5054. doi: 10.1038/s41467-024-48728-w (PMC11176386; doi:10.1038/s41467-024-48728-w)
Supplement: Supplementary file 1 — Supplementary Information [file 41467_2024_48728_MOESM1_ESM.pdf]

Supplementary information

Photochromic luminescence of organic crystals arising from subtle molecular rearrangement

Zihao Zhao[1], Yusong Cai[1], Qiang Zhang[1], Anze Li[1], Tianwen Zhu[1], Xiaohong Chen[1] & Wang Zhang Yuan[1]*

[1]School of Chemistry and Chemical Engineering, Frontiers Science Center for Transformative Molecules, Shanghai Jiao Tong University, No. 800 Dongchuan Rd., Minhang District, Shanghai 200240, China
✉ e-mail: wzhyuan@sjtu.edu.cn

Table of contents

Supplementary Methods

Supplementary Figures and Tables

Supplementary References

Supplementary Methods

Reagents and materials. TPA (> 99.0%), MMTPA (> 98.0%), terephthaloyl chloride (> 99.0%) and ammonium persulfate (> 99.0 %) were purchased from Tokyo Chemical Industry (TCI) Co., Ltd. DMNDCA (> 99.0%) was purchased from Shanghai Aladdin Co., Ltd. Methanol (> 99.9%), ethanol (> 99.5%), 2-propanol (> 99.5%), triethylamine (> 99.5%), dichloromethane (DCM), ethyl acetate (EA), *n*-hexane, magnesium sulfate ($MgSO_4$, > 98.0%), acrylamide (> 99.0 %) and *N*,*N′*-methylenebisacrylamide (> 99.0%) were obtained from Shanghai Titan Co., Ltd. Methyl 4-(aminocarbonyl)benzoate (> 96.0%) was purchased from Beijing Innochem Co., Ltd. Dimethyl sulfoxide (DMSO) was obtained from Sinopharm Group Co., Ltd. 2-methyltetrahydrofuran (2-MTHF) (99%, SuperDry, stabilizer free) was purchased from J&K Scientific Co., Ltd. Pure water was bought from Hangzhou Wahaha Group Co., Ltd (Zhejiang, China). All reagents for photoluminescence measurements were purified by recrystallization and/or column chromatography before use to guarantee their purity.

Instrumentation. ^{1}H and ^{13}C NMR spectra were obtained from a Bruker Avance III HD 500 MHz NMR spectrometer. EPR spectra were measured on a Bruker EMX-8 spectrometer. High-performance liquid chromatography (HPLC) was performed on a Waters Acquity UPLC/FLR/SQD2. Prompt and delayed emission spectra, quantum efficiencies and lifetimes were measured on an Edinburgh FLS1000 photoluminescence spectrometer. XRD analyses were performed on a Bruker D8 ADVANCE Da Vinci diffractometer. FTIR spectra were recorded on a Thermo Nicolet iN10 MX infrared imaging microscope. Raman spectra were measured with a Renishaw inVia Qontor confocal Raman microscope. Single-crystal structures were collected on a Bruker D8 VENTURE CMOS Photon II X-ray diffractometer with helios mx multilayer monochrmator Cu Ka radiation (λ = 1.54178 Å). Data collection, unit cell refinement and data reduction were performed using APEX3 v2019.11-0. The structure was solved by Intrinsic Phasing method and refined by full-matrix least-squares on F2 with anisotropic displacement parameters for the non-H atoms using SHELXTL program package. The hydrogen atoms on carbon were calculated in ideal positions with isotropic displacement parameters set to 1.2×Ueq of the attached atom (1.5×Ueq for methyl hydrogen atoms). The hydrogen atoms bound to nitrogen were located in a ΔF map and refined with isotropic displacement parameters. All photographs and videos were taken by a digital camera (Sony α7sII, Japan).

Note: Unless specified, all characterizations were carried out under ambient conditions.

Synthesis of dimethyl terephthalate (DMTPA). 1.7 mL of triethylamine (Et_3N), 0.49 mL of methanol and 10 mL of dichloromethane (DCM) were separately added into a 50 mL round-bottom flask. Afterwards, 2.03 g of terephthaloyl chloride dissolved in 5 mL of DCM was added dropwise into the flask under N_2 through a syringe under vigorous stirring, when the system was cooled to 0 °C by ice-water bath. The mixture was then heated to room temperature and reacted for 2 h, monitored by TLC. After quenched by 10 mL of pure water, the organic phase was separated, washed with brine three times and dried by anhydrous magnesium sulfate overnight. After the removal of the solvent with a rotary evaporator under reduced pressure, the crude product was purified by column chromatography with *n*-hexane/ethyl acetate (EA) (v/v = 6/1) as eluent. A withe solid was obtained in 93.8% yield (1.82 g) after being dried in vacuum at 40 °C overnight. Single crystals for further characterization were obtained by slowly cooling its hot and saturated EA solution. ^{1}H NMR (500 MHz, $CDCl_3$) δ (ppm) 8.10 (s, 4H), 3.94 (s, 6H) (Supplementary Fig. 1). ^{13}C NMR (125 MHz, $CDCl_3$) δ (ppm) 166.42, 134.04, 129.69, 52.57 (Supplementary Fig. 2). The purity of DMTPA is proved by HPLC analysis (Supplementary Fig. 18).

Synthesis of diethyl terephthalate (DETPA). DETPA was synthesized via the similar procedures to those of DMTPA described above. A white solid was obtained in 92.7% yield. Single crystals for further characterization were obtained by slowly cooling its hot and saturated EA solution. ^{1}H NMR (500 MHz, $CDCl_3$) δ (ppm) 8.10 (s, 4H), 4.40 (q, *J* = 7.1 Hz, 4H), 1.41 (t, *J* = 7.1 Hz, 6H) (Supplementary Fig. 3). ^{13}C NMR (125 MHz, $CDCl_3$) δ (ppm) 165.98, 134.30, 129.59, 61.53, 14.41 (Supplementary Fig. 4). The purity of DETPA is proved by HPLC analysis (Supplementary Fig. 38).

Synthesis of diisopropyl terephthalate (DiPTPA). DiPTPA was synthesized via the similar procedures to those of DMTPA described above. A white solid was obtained in 95.2% yield. Single crystals of DiPTPA for further characterization were obtained by slowly cooling its hot and saturated EA solution. ^{1}H NMR (500 MHz, DMSO-d_6) δ (ppm) 8.05 (s, 4H), 5.16 (sep, *J* = 6.2 Hz, 2H), 1.33 (d, *J* = 6.3 Hz, 12H) (Supplementary Fig. 5). ^{13}C NMR (125 MHz, DMSO-d_6) δ (ppm) 164.48, 133.98, 129.31, 68.77, 21.56 (Supplementary Fig. 6). The purity of DiPTPA is proved by HPLC analysis (Supplementary Fig. 39).

Purification of 2,6-dimethyl 2,6-naphthalenedicarboxylate (DMNDCA). Commercially available DMNDCA was firstly purified by column chromatography with *n*-hexane/EA (v/v = 6/1) as eluent and dried in vacuum at 40 °C overnight. The obtained solid was further purified by recrystallization through slowly cooling its hot and saturated EA solution. The resulting crystals were collected through filtration and then dissolved in EA for further purification and single crystal cultivation. Single crystals were obtained by slowly evaporating its dilute EA solution at room temperature. ^{1}H NMR (500 MHz, $CDCl_3$) δ (ppm) 8.63 (s, 2H), 8.12 (d, *J* = 8.5 Hz, 2H), 8.00 (d, *J* = 8.6 Hz, 2H), 4.00 (s, *J* = 1.2 Hz, 6H) (Supplementary Fig. 7). ^{13}C NMR (125 MHz, $CDCl_3$) δ (ppm) 167.00, 134.73, 130.80, 129.73, 129.66, 126.16, 52.58 (Supplementary Fig. 8).

Purification of monomethyl terephthalate (MMTPA). Commercially available MMTPA was firstly purified by column chromatography with DCM/EA (v/v = 1/1) as eluent and dried in vacuum at 40 °C overnight. The obtained MMTPA was further purified by recrystallization performed through slowly cooling its hot and saturated EA/methanol solution. The resulting crystals were collected through filtration and then dissolved in EA for further purification and single crystal cultivation. Single crystals of MMTPA were obtained by slowly evaporating its dilute EA solution at room temperature. ^{1}H NMR (500 MHz, DMSO-d_6) δ (ppm) 13.34 (s, 1H), 8.05 (d, J = 2.4 Hz, 4H), 3.88 (s, 3H) (Supplementary Fig. 9). ^{13}C NMR (125 MHz, DMSO-d_6) δ (ppm) 166.57, 165.62, 134.82, 133.17, 129.61, 129.36, 52.46 (Supplementary Fig. 10). The purity of MMTPA is proved by HPLC analysis (Supplementary Fig. 66).

Purification of terephthalic acid (TPA). Commercially available TPA was firstly purified by RP-C18 column chromatography and dried in vacuum at 40 °C overnight. The obtained TPA was further purified by slowly evaporating its dilute dimethyl sulfoxide (DMSO) solution at 30 °C. Unfortunately, we failed to acquire TPA single crystals due to the excessively strong interactions among the molecules. The powder crystals were obtained instead, whose crystalline nature is verified by the sharp and intense peaks in XRD patterns. ^{1}H NMR (500 MHz, DMSO-d_6) δ (ppm) 13.29 (s, 2H), 8.04 (s, 4H) (Supplementary Fig. 11). ^{13}C NMR (125 MHz, DMSO-d_6) δ (ppm) 167.14, 134.90, 129.92 (Supplementary Fig. 12).

Purification of methyl 4-(aminocarbonyl)benzoate. Commercially available methyl 4-(aminocarbonyl)benzoate was purified by recrystallization performed through slowly cooling its hot and saturated EA solution. The resulting crystals were collected through filtration and then dissolved in EA for further purification and single crystal cultivation. Single crystals were obtained by slowly evaporating its dilute EA solution at room temperature. ^{1}H NMR (500 MHz, DMSO-d_6) δ (ppm) 8.14 (s, 1H), 8.05 – 7.95 (m, 4H), 7.56 (s, 1H), 3.88 (s, 3H) (Supplementary Fig. 13). ^{13}C NMR (125 MHz, DMSO-d_6) δ (ppm) 167.03, 165.75, 138.44, 131.80, 129.07, 127.83, 52.37 (Supplementary Fig. 14).

Supplementary Figures and Tables

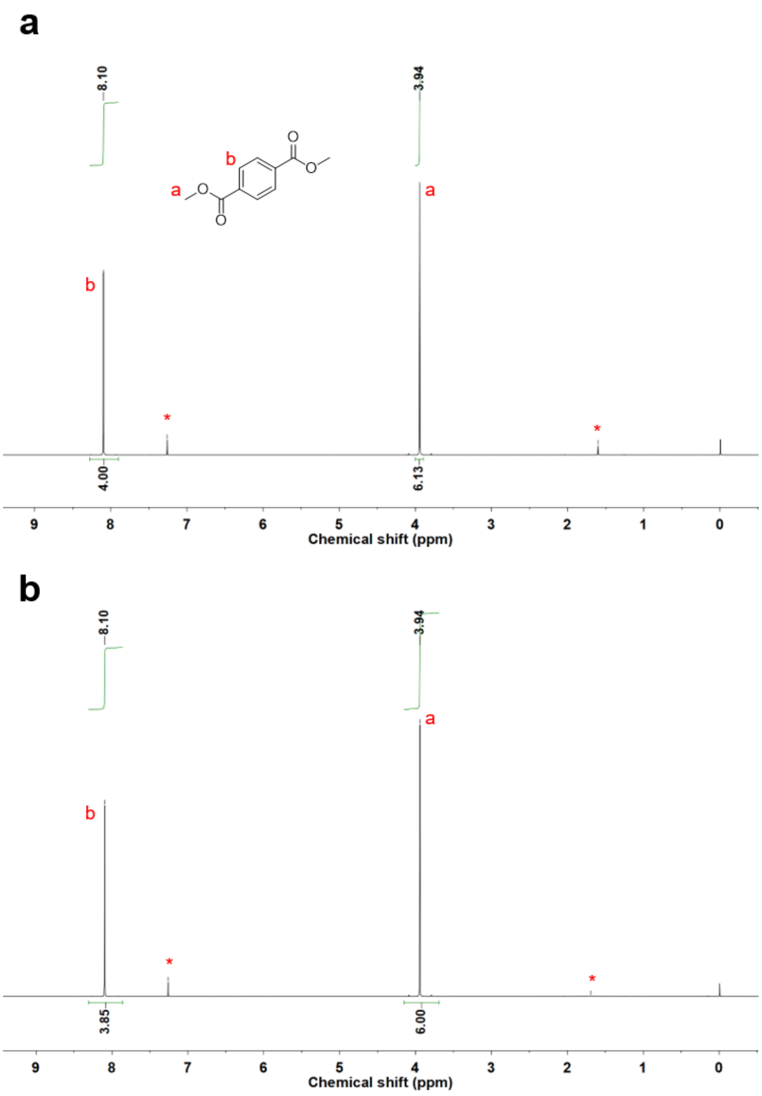

Supplementary Fig. 1. ^{1}H NMR characterization of DMTPA. ^{1}H NMR spectra of DMTPA (a) before and (b) after 12 h of 312 nm UV irradiation.

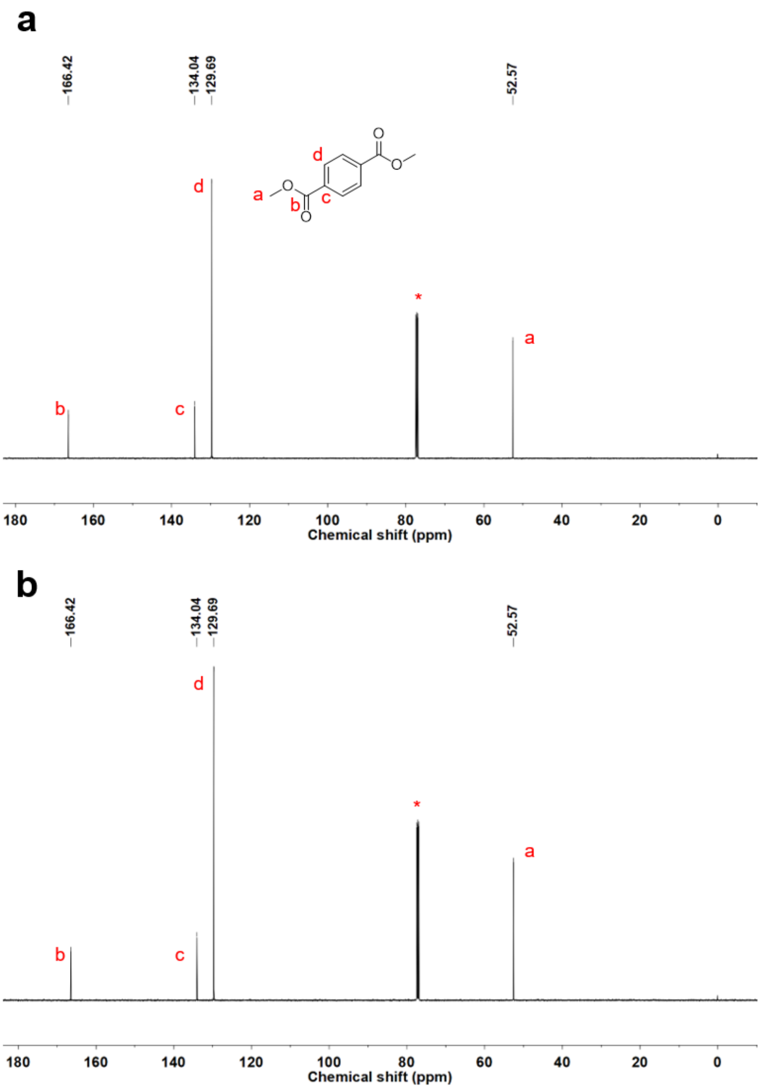

Supplementary Fig. 2. ^{13}C NMR characterization of DMTPA. ^{13}C NMR spectra of DMTPA (a) before and (b) after 12 h of 312 nm UV irradiation.

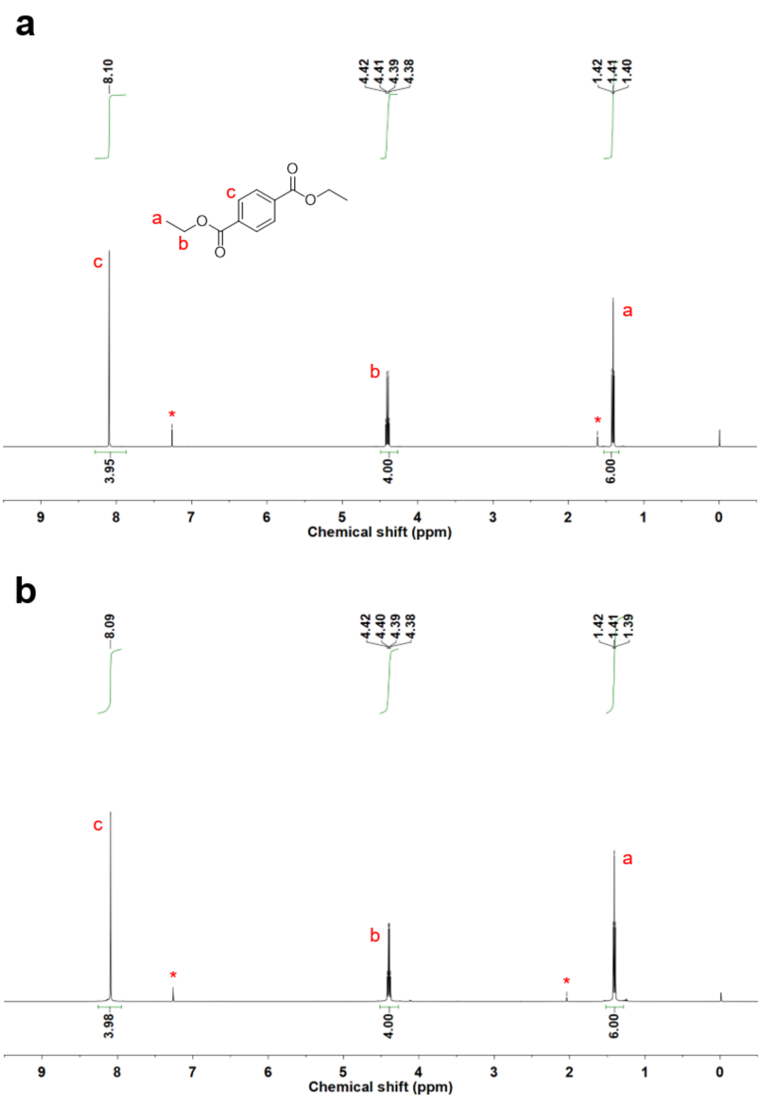

Supplementary Fig. 3. ^{1}H NMR characterization of DETPA. ^{1}H NMR spectra of DETPA (a) before and (b) after 12 h of 312 nm UV irradiation.

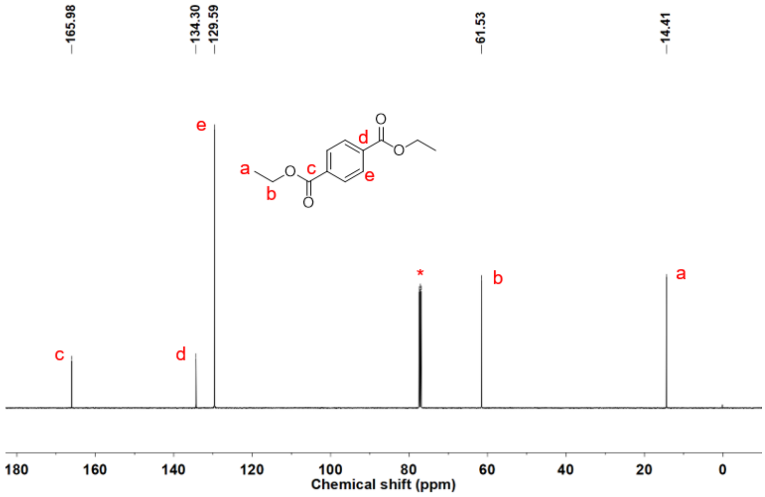

Supplementary Fig. 4. ^{13}C NMR spectra of DETPA.

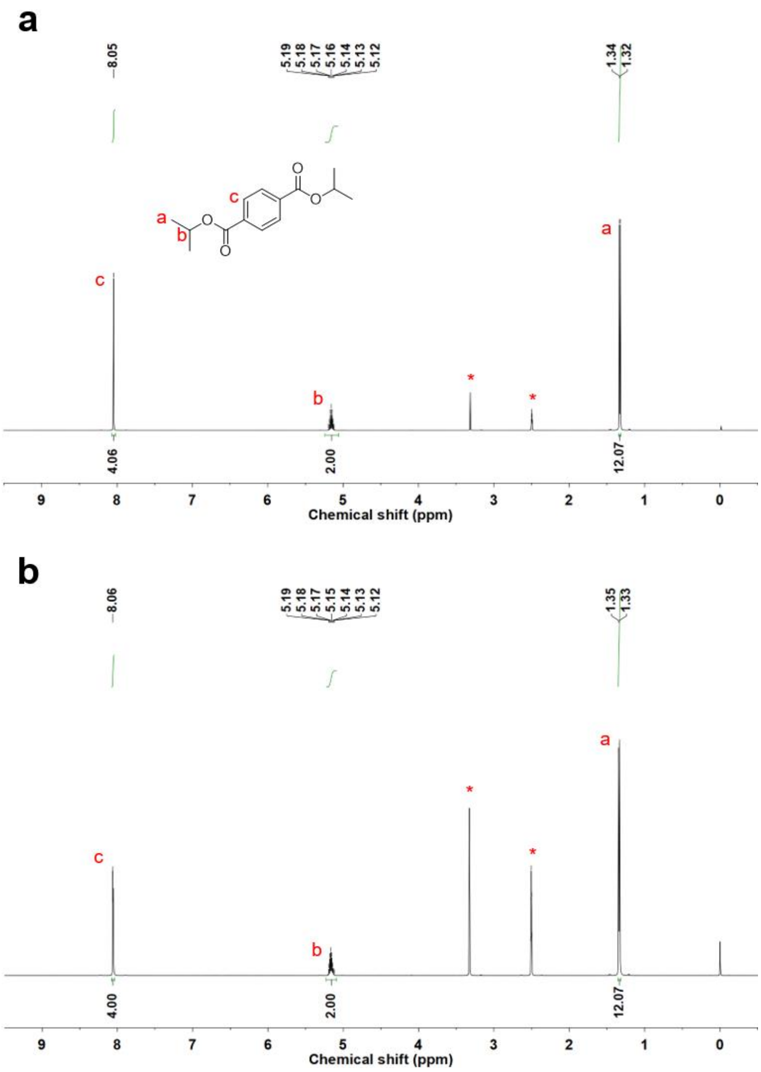

Supplementary Fig. 5. ^{1}H NMR characterization of DiPTPA. ^{1}H NMR spectra of DiPTPA (a) before and (b) after 12 h of 312 nm UV irradiation.

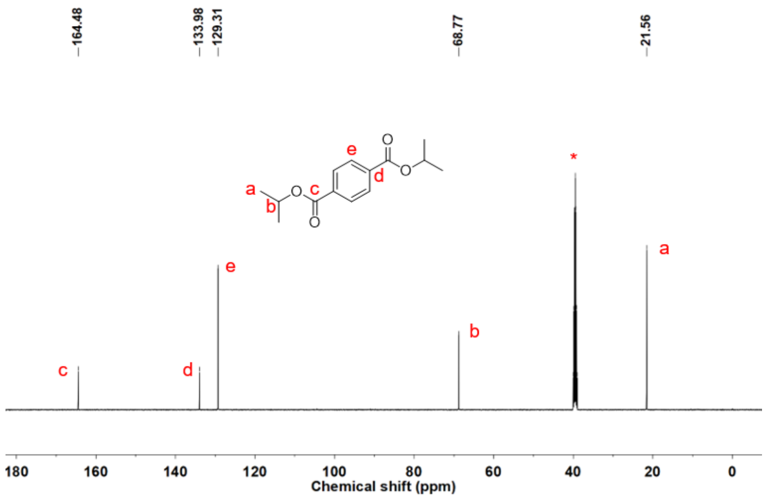

Supplementary Fig. 6. ^{13}C NMR spectra of DiPTPA.

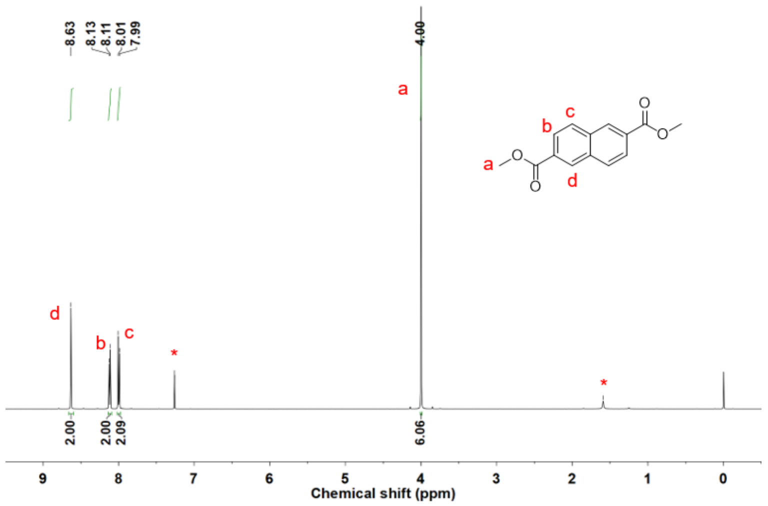

Supplementary Fig. 7. ^{1}H NMR spectra of DMNDCA.

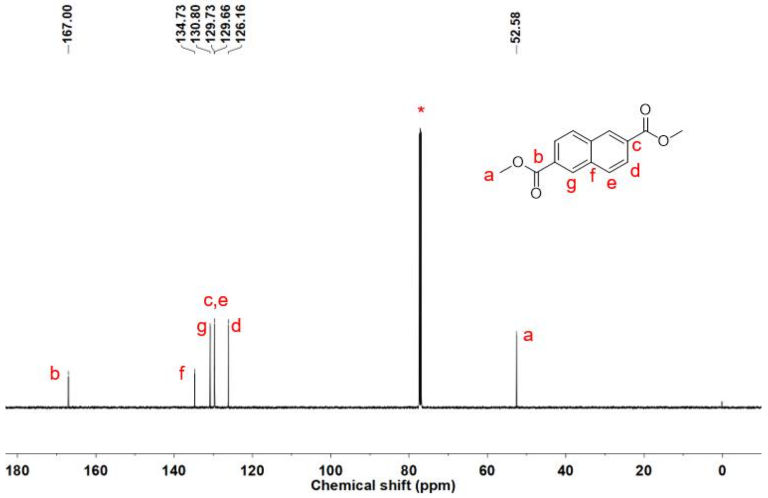

Supplementary Fig. 8. ^{13}C NMR spectra of DMNDCA.

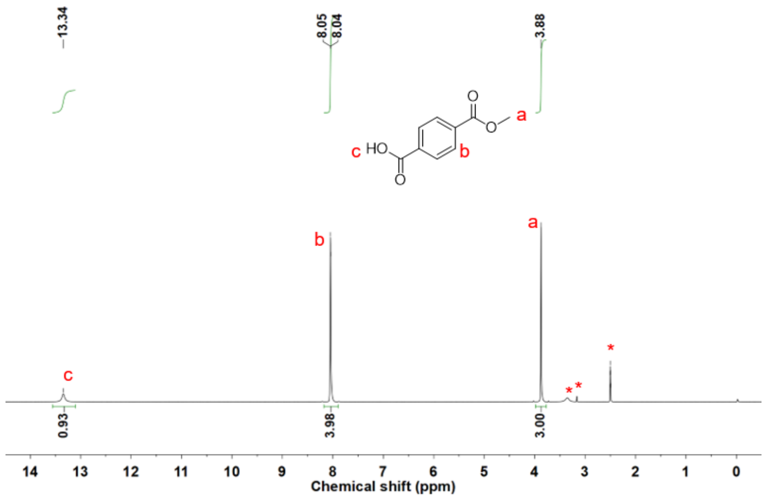

Supplementary Fig. 9. ^{1}H NMR spectra of MMTPA.

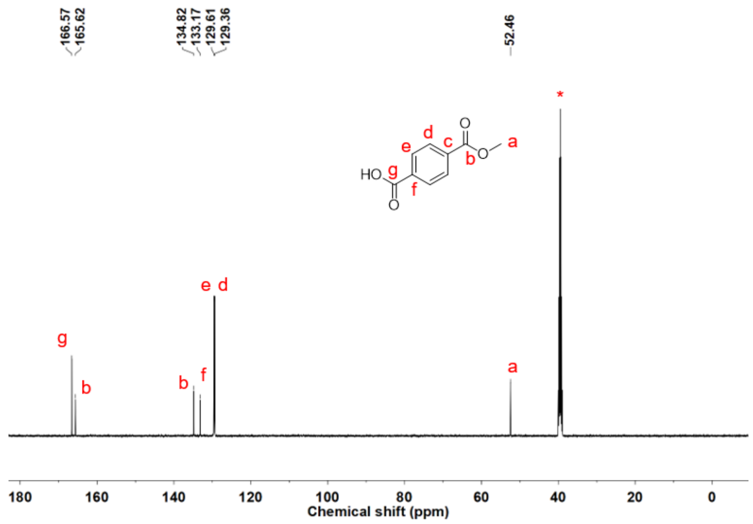

Supplementary Fig. 10. ^{13}C NMR spectra of MMTPA.

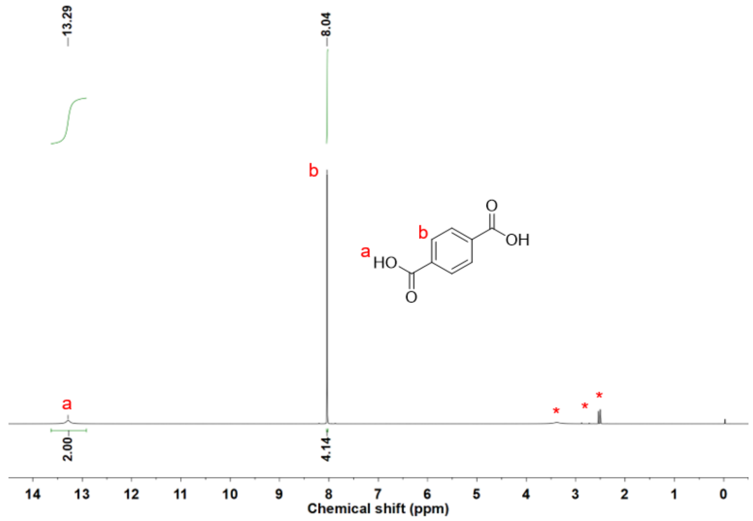

Supplementary Fig. 11. ^{1}H NMR spectra of TPA.

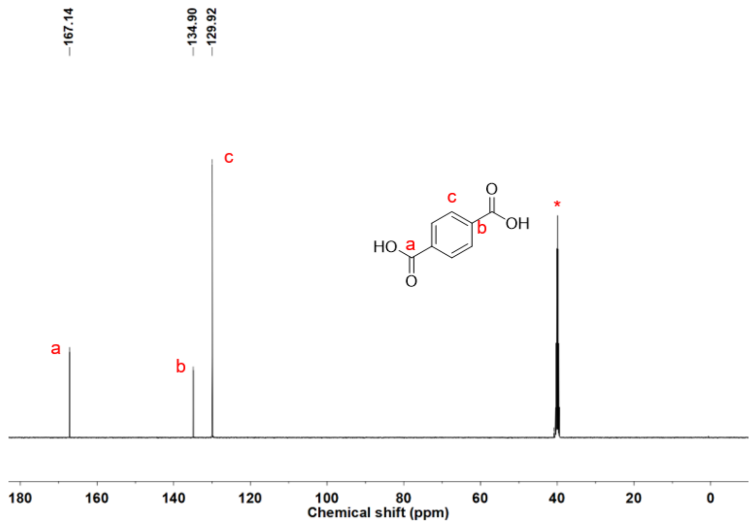

Supplementary Fig. 12. ^{13}C NMR spectra of TPA.

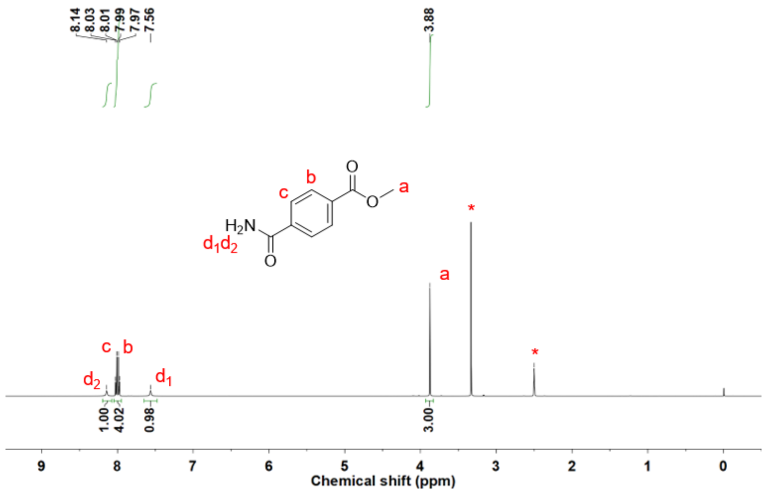

Supplementary Fig. 13. ^{1}H NMR spectra of methyl 4-(aminocarbonyl)benzoate.

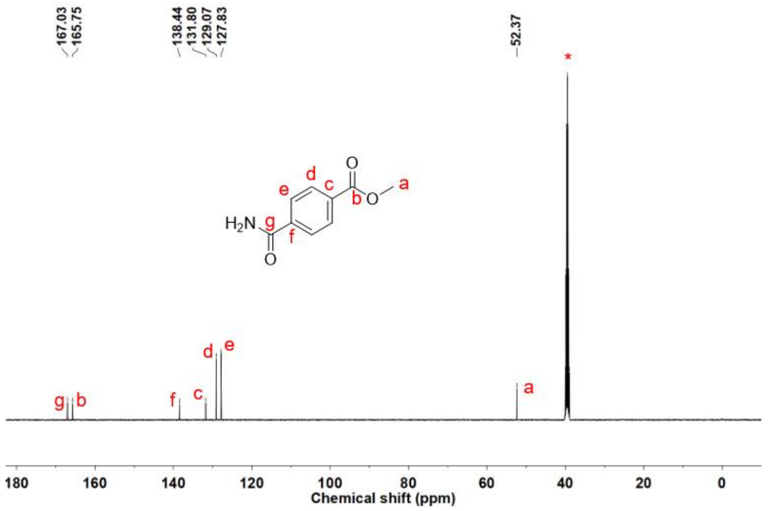

Supplementary Fig. 14. ^{13}C NMR spectra of methyl 4-(aminocarbonyl)benzoate.

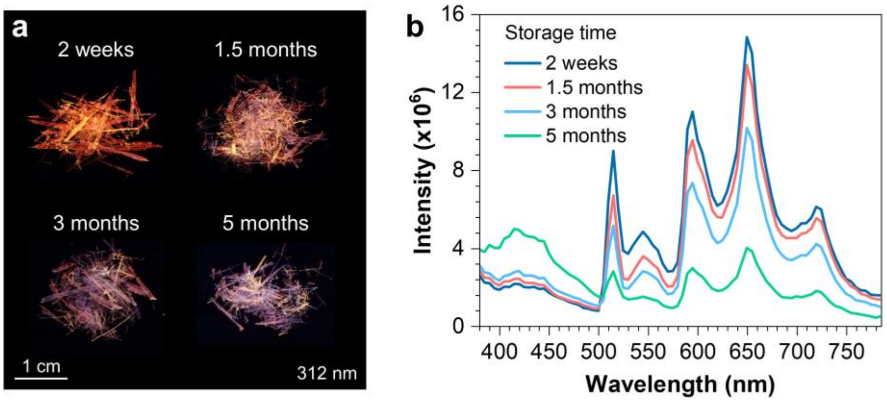

Supplementary Fig. 15. Stability of newly generated long-wavelength emissive species. (a) Luminescent photographs and (b) emission spectra of DMTPA crystals placed under ambient conditions for different time (λ_{ex} = 312 nm).

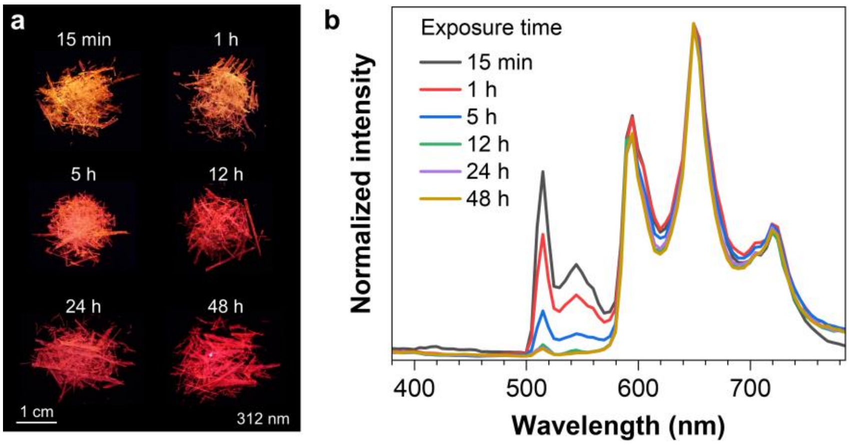

Supplementary Fig. 16. PCL process under longer irradiation time. (a) Luminescent photographs and (b) emission spectra of DMTPA crystals under 312 nm UV irradiation with different exposure time (> 15 min).

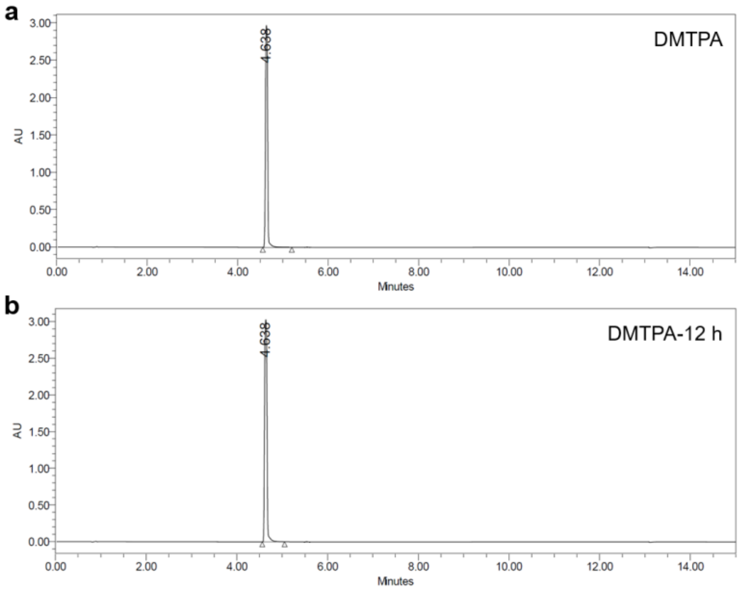

Supplementary Fig. 17. HPLC analysis for DMTPA. HPLC results for DMTPA crystals (a) before and (b) after 12 h of 312 nm UV irradiation. The y-axis unit AU represents absorbance unit.

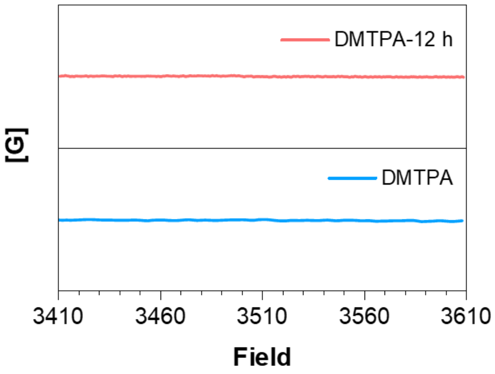

Supplementary Fig. 18. EPR results for DMTPA. EPR spectra of DMTPA crystals before and after 12 h of 312 nm UV irradiation.

Supplementary Note 1. To accumulate the products of the possible photochemical reactions, DMTPA crystals were irradiated by 312 nm UV light for 12 h before corresponding NMR, EPR and HPLC analyses, whose results show no difference from those of unstimulated DMTPA crystals, suggesting the PL transformation is not caused by photochemical changes on DMTPA molecules.

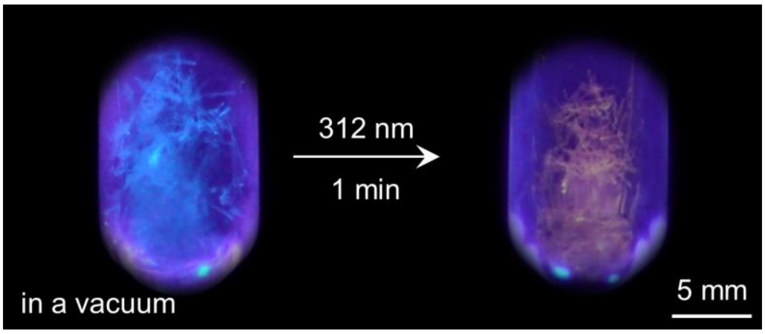

Supplementary Fig. 19. PCL process in a vacuum. The luminescent photographs of PL colour transformation process of DMTPA crystals in a vacuum under 312 nm UV irradiation.

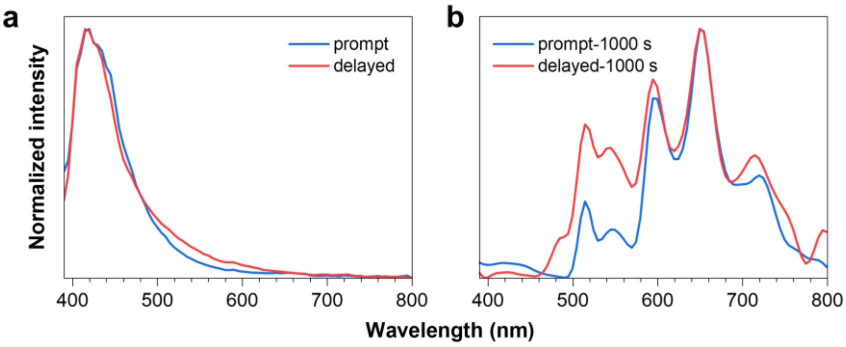

Supplementary Fig. 20. Comparison of prompt and delayed emission spectra of DMTPA crystals. Prompt and delayed (t_d = 1 ms) emission spectra of DMTPA crystals (a) before and (b) after 1000 s of 312 nm UV irradiation (λ_{ex} = 312 nm).

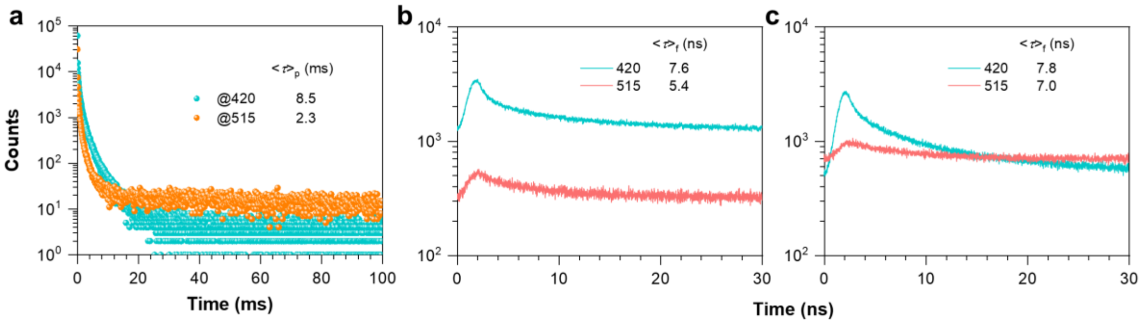

Supplementary Fig. 21. Lifetime profiles of DMTPA crystals. (a) Millisecond-scale lifetimes for DMTPA crystals before UV irradiation (λ_{ex} = 312 nm). Nanosecond-scale lifetimes for DMTPA crystals (b) before and (c) after 312 nm UV irradiation (λ_{ex} = 312 nm). Except those at 420 and 515 nm, no nanosecond-scale lifetimes can be detected at other peaks in emission spectra of DMTPA crystals.

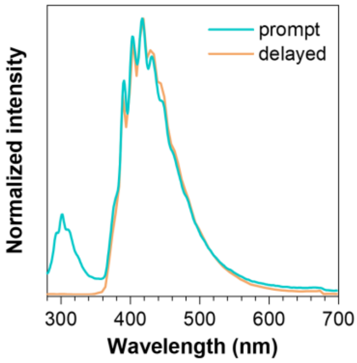

Supplementary Fig. 22. Photophysical properties of dilute DMTPA solution at 77 K. Prompt and delayed emission spectra of 10^{-5} M DMTPA/2-MTHF solution at 77 K (λ_{ex} = 312 nm).

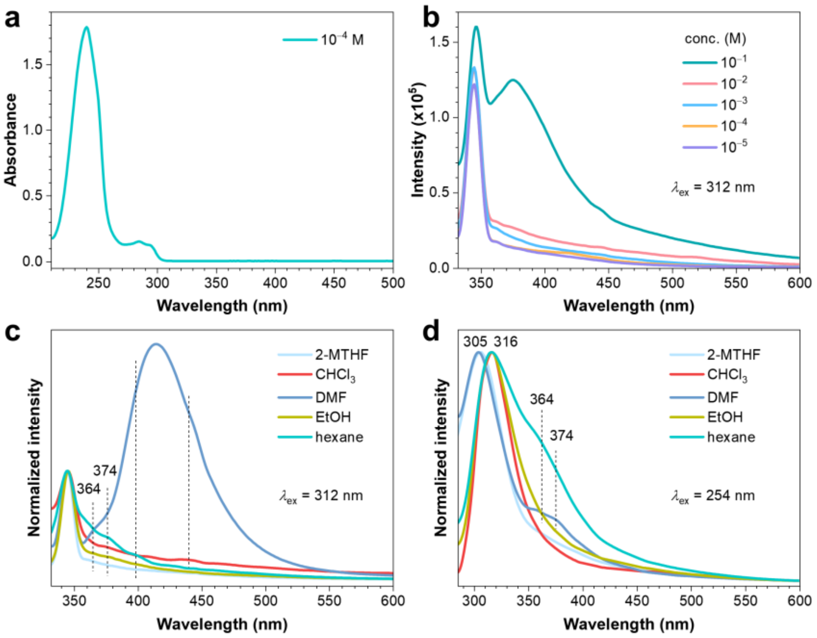

Supplementary Fig. 23. Photophysical properties of DMTPA solutions. (a) Absorption of 10^{-4} M DMTPA/2-MTHF solution. Emission spectra of (b) varying DMTPA/2-MTHF solutions, (c) 10^{-3} M and (d) 10^{-5} M DMTPA solutions with different solvents (λ_{ex} = 312 or 254 nm).

Supplementary Note 2. The UV-vis absorption of 10^{-4} M DMTPA/2-MTHF solution exhibits an evident E_2 band (240 nm) and a B band (285 nm) of benzene ring (Supplementary Fig. 23a). Except for the Raman peak at 345 nm, the concentrated (10^{-1} M) DMTPA/2-MTHF solution demonstrates emission centred at 374 nm with considerable PL intensity, which drastically attenuates as the concentration decreases to 10^{-2} M and lower, illustrating a concentration-enhanced emission characteristic (Supplementary Fig. 23b). The emission peak and shoulder at 374 and 364 nm should be attributed to DMPTA excimers, while the shoulder at ~440 nm along with tails extended to much longer wavelength region should be attributed to various DMTPA clusters. According to the clustering-triggered emission (CTE) mechanism, DMTPA molecules are discrete in dilute solutions, while efficiently clusters with each other in concentrated counterparts. Therefore, in concentrated solutions, the original limited molecular conjugation is effectively extended, in favour of emission, and the severe nonradiative deactivations can be restrained. Furthermore, when dissolved in different solvents, DMTPA solutions (10^{-3} M) show diverse emission spectra (Supplementary Fig. 23c). The excimer peaks (364 and 374 nm) are observed in dilute DMTPA/DMF and DMTPA/hexane solutions (10^{-5} M), while absent in DMTPA/2-MTHF, DMTPA/$CHCl_3$ and DMTPA/EtOH counterparts (Supplementary Fig. 23d). Various polarity and solubility endow DMTPA shows different degrees of dispersion in these solvents, leading to distinct emission spectra.

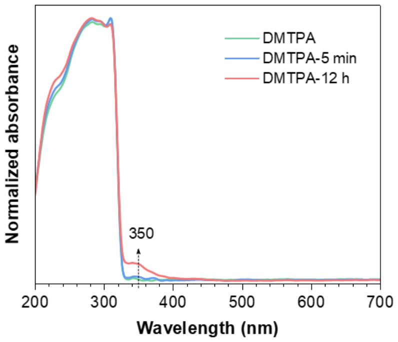

Supplementary Fig. 24. Absorption of DMTPA crystals. Absorption of DMTPA crystals before and after 5 min and 12 h of 312 nm UV irradiation.

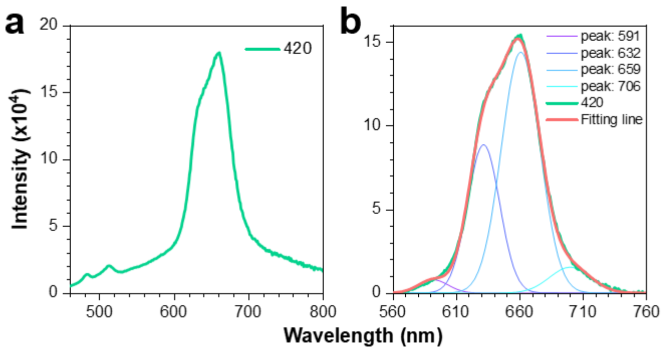

Supplementary Fig. 25. Deconvolution of DMTPA emission spectrum. (a) Emission spectrum and (b) corresponding multi-peaks Gaussian fitting of DMTPA crystals after 1000 s of 312 nm UV irradiation (λ_{ex} = 420 nm).

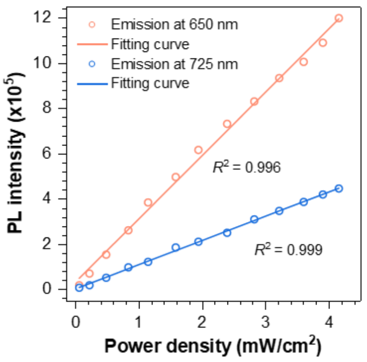

Supplementary Fig. 26. The linear relationship between PL intensity and power density. Plots of PL intensity (λ_{ex} = 650 and 725 nm) as a function of excitation power density (λ_{ex} = 312 nm).

Supplementary Note 3. Because of the finite concentration and lifetime of permanent lattice defect, the PL intensity of excitons bound to or associated with defects would be saturated at high excitation power density. However, a linear dependence on the excitation power density is observed from the PL intensity of photogenerated peaks (*e.g.* 650 and 725 nm) of DMTPA crystals after 312 nm UV irradiation, indicating the emission is originated from photogenerated excitons rather than the photoinduced generation of clusters in lattice defects.

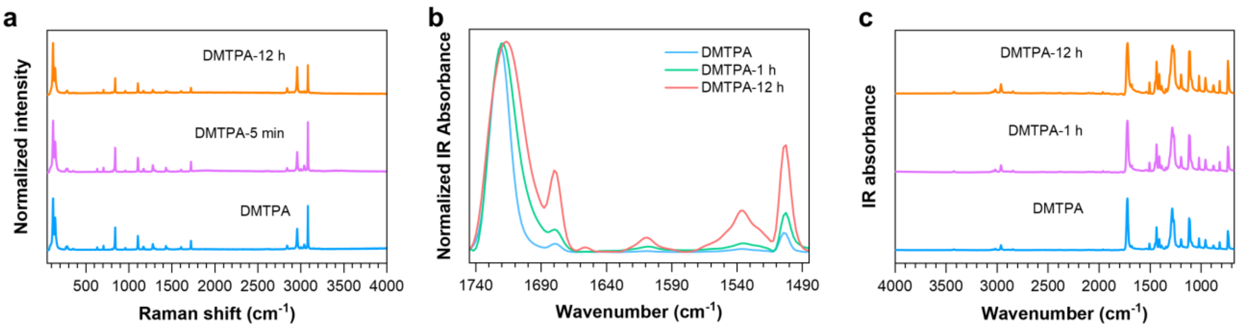

Supplementary Fig. 27. Raman and FTIR characterizations of DMTPA crystals. (a) The whole range of the Raman spectra. (b) The local and (c) the whole range of the FTIR spectra of DMTPA crystals with different exposure time under 312 nm UV irradiation.

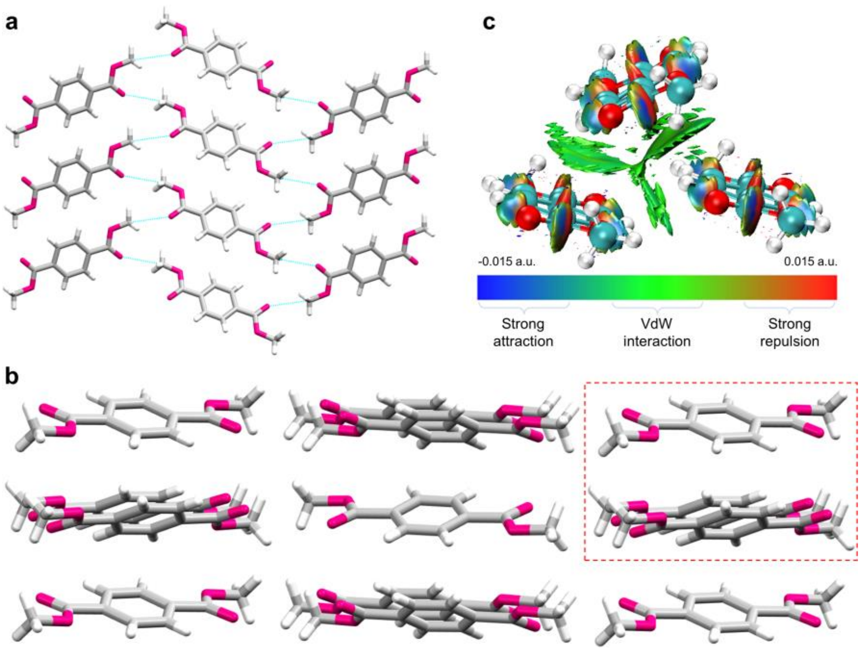

Supplementary Fig. 28. Single crystal analysis of DMTPA. (a, b) Single crystal structure and fragmental molecular packing of DMTPA. (c) The noncovalent interactions (NCI) plot of DMTPA trimer marked by dashed box in (b). The green area among DMTPA molecules implies the widespread edge-to-face π-π interactions.

Supplementary Note 4. Noncovalent interactions (NCI) analysis can be used to visualize secondary bonds, such as hydrogen bonds and van der Waals (VDW) interactions[1,2]. Labelled in different colours, the strong repulsion, VDW interactions and strong attractions are identified clearly in NCI plots. Therefore, the green area in Supplementary Fig. 10c indicates obvious edge-to-face π-π interactions among the DMTPA trimer, which can construct a widespread through-space conjugation (TSC) network throughout the crystal lattice.

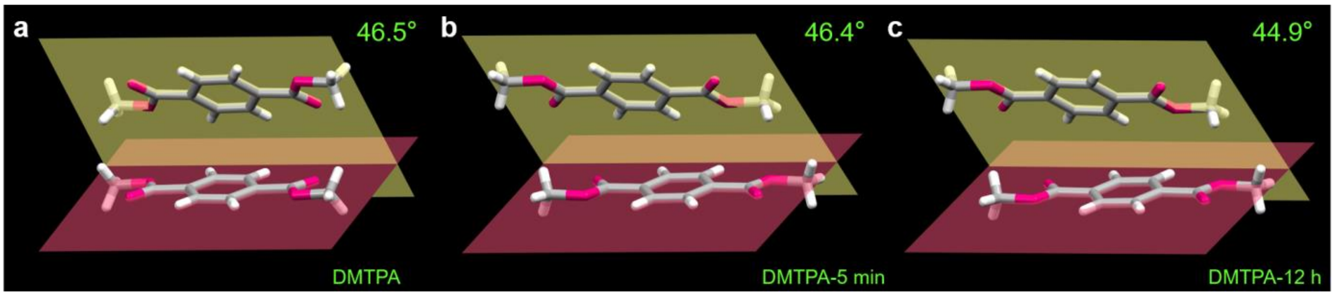

Supplementary Fig. 29. Evolution of dihedral angle in DMTPA crystals. The transformation of dihedral angle between two DMTPA molecules in crystal (a) before and after (b) 5 min and (c) 12 h of 312 nm UV irradiation.

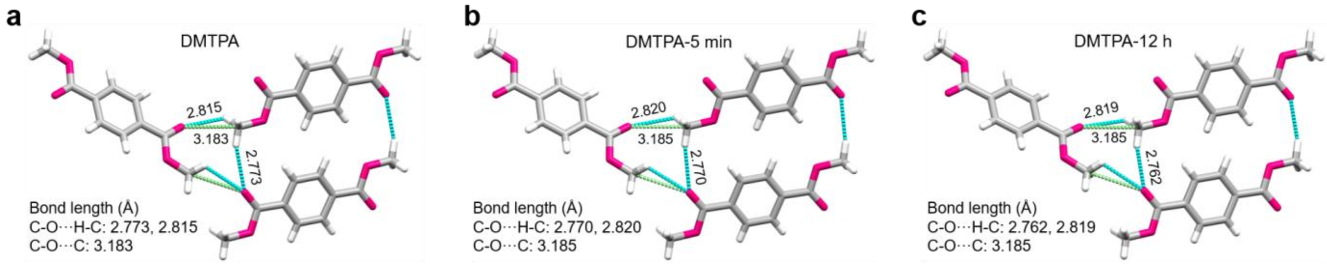

Supplementary Fig. 30. Evolution of bond lengths in DMTPA crystals. The transformation of bond lengths of denoted intermolecular interactions in DMTPA crystal (a) before and after (b) 5 min and (c) 12 h of 312 nm UV irradiation.

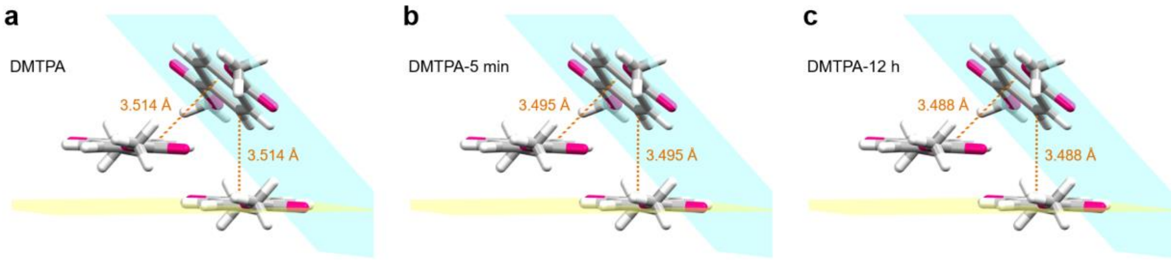

Supplementary Fig. 31. Evolution of molecular distances in DMTPA crystals. The transformation of distances between DMTPA molecules with edge-to-face π-π interactions in crystal (a) before and after (b) 5 min and (c) 12 h of 312 nm UV irradiation.

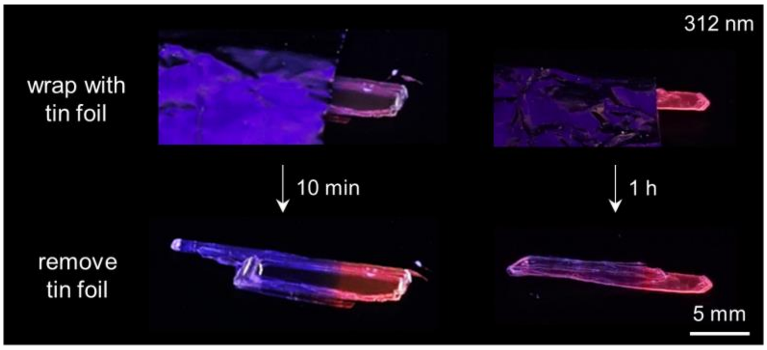

Supplementary Fig. 32. PCL process upon local irradiation. Luminescent photographs of DMTPA crystals after locally irradiated by 312 nm UV light with different exposure time.

Supplementary Note 5. Part of the bulk DMTPA crystal was wrapped in aluminium foil to block the 312 nm UV irradiation (Supplementary Fig. 32). After 10 min of irradiation, the irradiated part demonstrated orange PL while the covered area, after removing the aluminium foil, still retained the blue emission. The small region at the boundary of the irradiated and covered part exhibited purple PL, exact the intermediate state of the photochromic luminescence (PCL) process. To investigate whether this phenomenon only happens at the intersection or can be propagated to the deeper site of the covered part of crystal, we prolonged the irradiation time to facilitate the completion of possible propagation process. After 1 h of irradiation and removal of the aluminium foil, the covered region was completely turn into purple emission. These results indicate the PCL process can be propagated across the crystal from the irradiated point. This could also provide extra evidence for the proposed amplification effect.

Supplementary Table 1. Single crystal data of DMTPA before and after 12 h of 312 nm UV irradiation.

	DMTPA	DMTPA-12 h
Formula	$C_{10}H_{10}O_4$	$C_{10}H_{10}O_4$
Formula Weight	194.18	194.18
Wavelength (Å)	1.54178	0.70173
Space Group	Pbca	Pbca
Cell Length (Å)	a=7.1191 (7)<br>b=6.0236 (5)<br>c=21.9995 (15)	a=7.1131 (6)<br>b=6.0159 (5)<br>c=21.9960 (17)
Cell Angle (°)	α=90<br>β=90<br>γ=90	α=90<br>β=90<br>γ=90
Cell Volume (Å^3)	943.40 (14)	941.25 (13)
Z	4	4
Density (g/cm^3)	1.367	1.370
F (000)	408.0	408.0
h_{max}, k_{max}, l_{max}	8, 7, 26	8, 7, 26
T_{min}, T_{max}	0.806, 0.835	0.881, 0.914

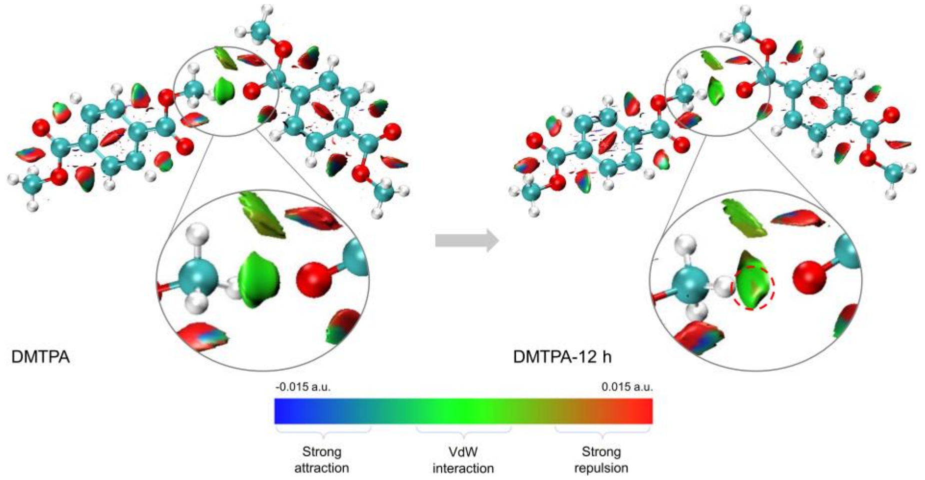

Supplementary Fig. 33. NCI analysis of DMTPA. The NCI plot of transversely arranged DMTPA molecules before and after 12 h of 312 nm UV irradiation. The small red area labelled by the dashed circle in DMTPA-12 h suggests the presence of repulsion between these two molecules after UV stimulus, which leads to the impairing of corresponding short contacts. The theoretically calculated result shows superior consistence with the above experimental results.

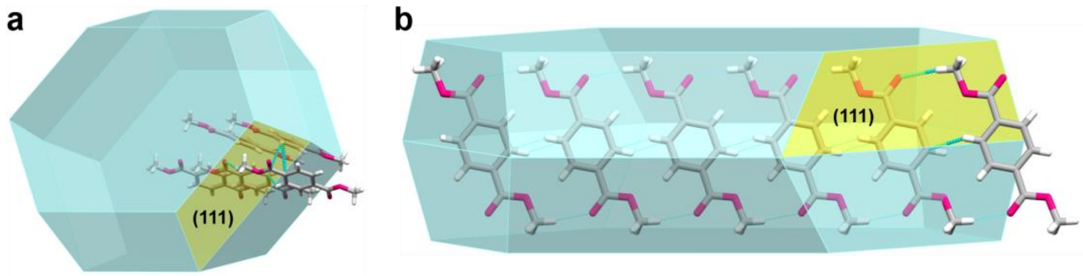

Supplementary Fig. 34. Crystal morphology of DMTPA. (a,b) Predicted crystal morphology and intermolecular interactions corresponding to crystal face (111).

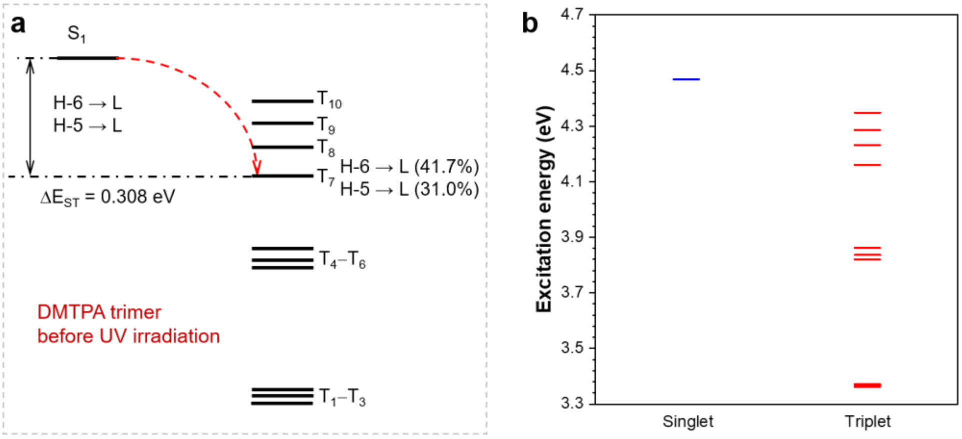

Supplementary Fig. 35. Calculated transition configurations of DMTPA trimer before UV irradiation. (a) The ISC channels and (b) excitation energy diagram of DMTPA trimer before exposed to 312 nm UV irradiation. H and L represent HOMO and LUMO, respectively.

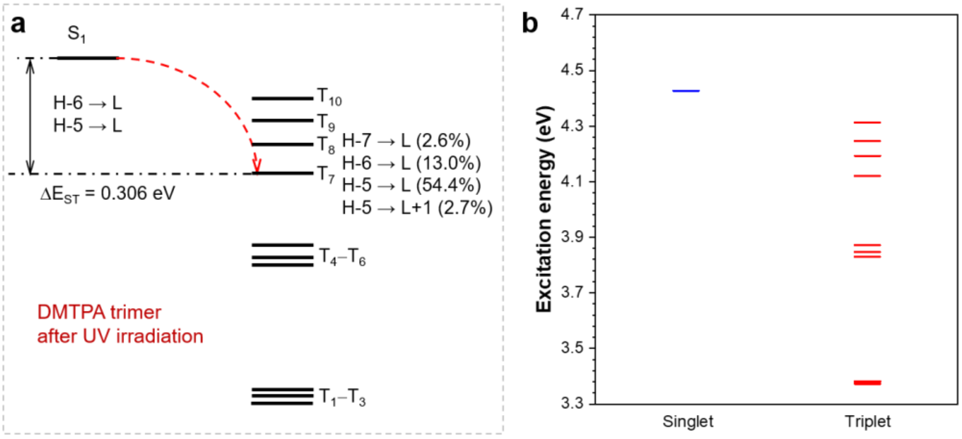

Supplementary Fig. 36. Calculated transition configurations of DMTPA trimer after UV irradiation. (a) The ISC channels and (b) excitation energy diagram of DMTPA trimer after 12 h of 312 nm UV irradiation. H and L represent HOMO and LUMO, respectively.

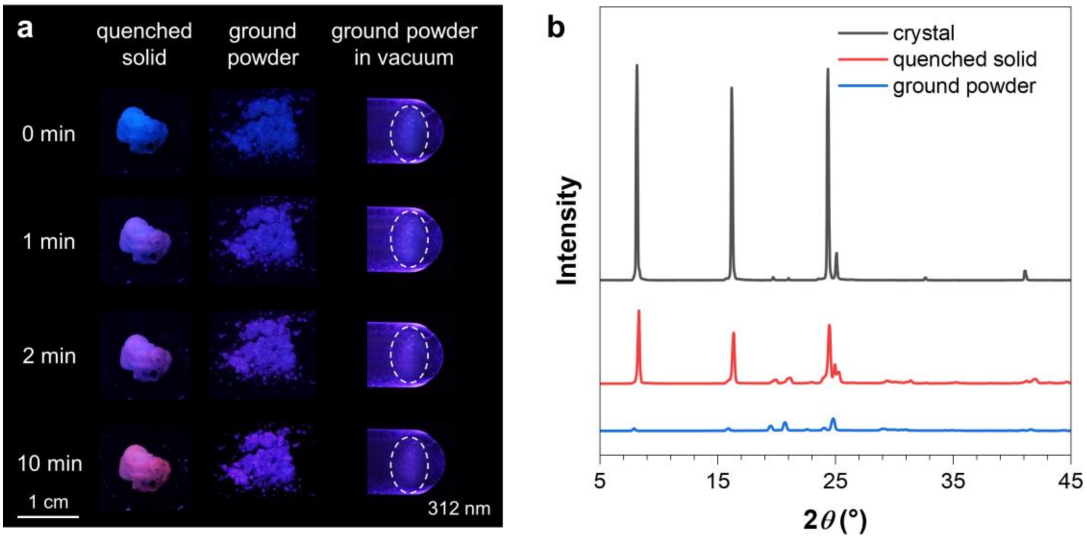

Supplementary Fig. 37. PCL process and XRD characterization of different DMTPA solids. (a) Luminescent photographs of liquid nitrogen quenched DMTPA solid and its ground powder under 312 nm UV irradiation with different exposure time. (b) XRD patterns of the crystals, liquid nitrogen quenched solid and ground powder of DMTPA.

Supplementary Note 6. Compact lattice structure is the prerequisite of dense and orderly periodic TSC network, as well as the subsequent amplification effect. To verify the necessity of crystal structure for PCL of these compounds, the liquid nitrogen quenched DMTPA solid and its ground powder were investigated. As Supplementary Fig. 37a shows, although the liquid nitrogen quenched DMTPA solid still exhibit PCL emission from blue to magenta under 312 nm UV irradiation, the range of PL variation becomes narrower compared with that of DMTPA crystals, together with slower response time. As the quenched solid is further ground into powders, the PCL phenomenon becomes inconspicuous. Notably, no obvious PCL behaviours can be noticed even when the ground powder is placed in vacuum. The XRD patterns of these samples reveal a gradual decrease in crystallization degree (Supplementary Fig. 37b), demonstrating the high consistency between PCL and the crystallinity of DMTPA. With the gradual destruction of lattice, the TSC network also collapses, thus leading to the attenuation of PCL performance.

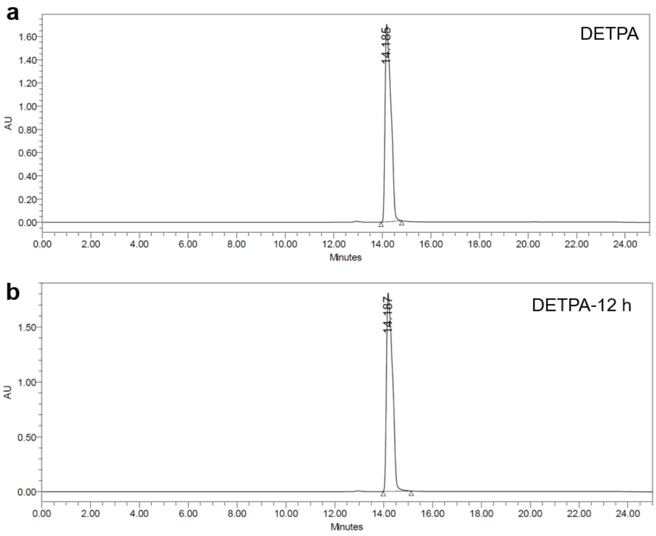

Supplementary Fig. 38. HPLC analysis for DETPA. HPLC results for DETPA crystals (a) before and (b) after 12 h of 312 nm UV irradiation. The y-axis unit AU represents absorbance unit.

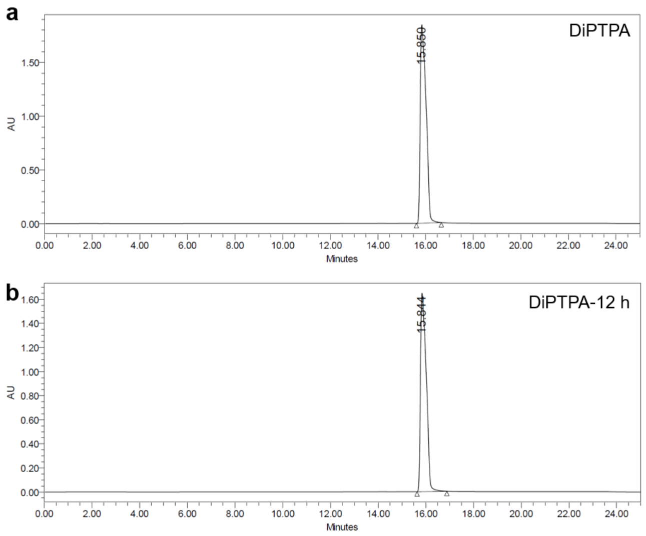

Supplementary Fig. 39. HPLC analysis for DiPTPA. HPLC results for DiPTPA crystals (a) before and (b) after 12 h of 312 nm UV irradiation. The y-axis unit AU represents absorbance unit.

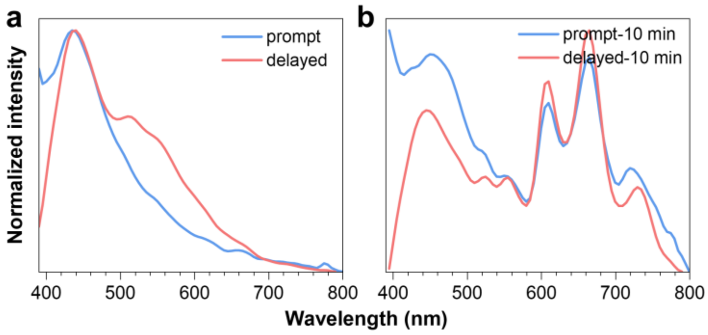

Supplementary Fig. 40. Comparison of prompt and delayed emission spectra of DETPA crystals. Prompt and delayed (t_d = 1 ms) emission spectra of DETPA crystals (a) before and (b) after 10 min of 312 nm UV irradiation (λ_{ex} = 312 nm).

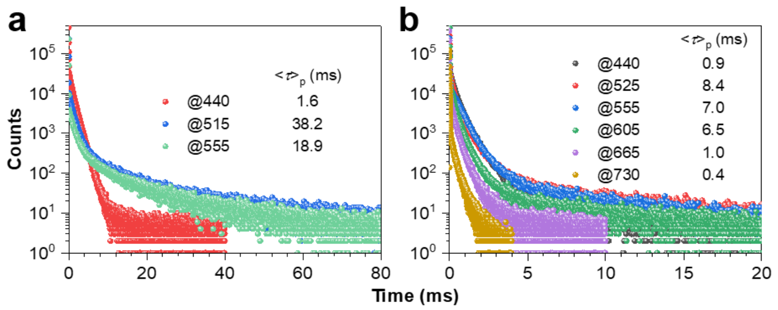

Supplementary Fig. 41. Millisecond-scale lifetime profiles of DETPA crystals. Millisecond-scale lifetimes for DETPA crystals (a) before and (b) after 312 nm UV irradiation (λ_{ex} = 312 nm).

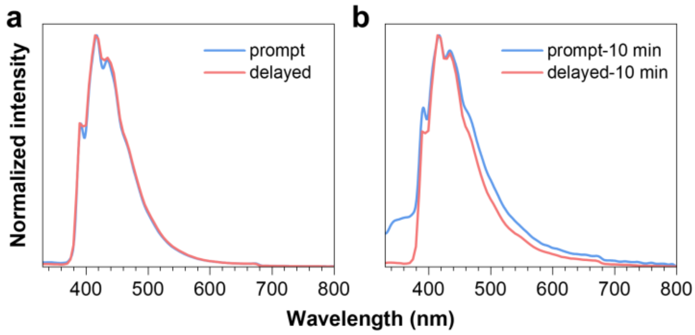

Supplementary Fig. 42. Comparison of prompt and delayed emission spectra of DiPTPA crystals. Prompt and delayed (t_d = 1 ms) emission spectra of DiPTPA crystals (a) before and (b) after 10 min of 312 nm UV irradiation (λ_{ex} = 312 nm).

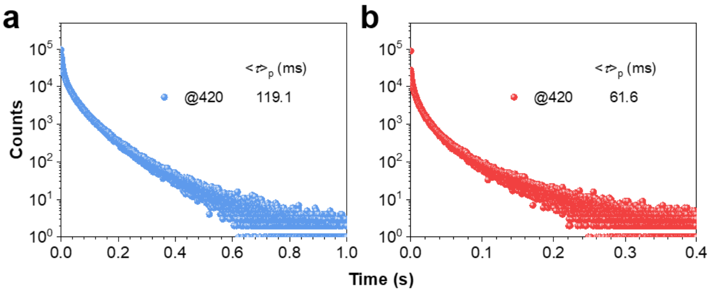

Supplementary Fig. 43. Millisecond-scale lifetime profiles of DiPTPA crystals. Millisecond-scale lifetimes for DiPTPA crystals (a) before and (b) after 312 nm UV irradiation (λ_{ex} = 312 nm).

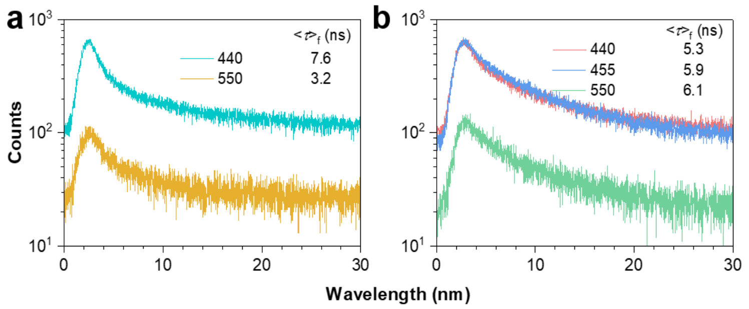

Supplementary Fig. 44. Nanosecond-scale lifetime profiles of DETPA crystals. Nanosecond-scale lifetime for DETPA crystals (a) before and (b) after 10 min of 312 nm UV irradiation (λ_{ex} = 312 nm). No nanosecond-scale lifetimes can be detected at other peaks in emission spectra of DETPA crystals. And no nanosecond-scale lifetimes can be measured at peaks shown in emission spectra of DiPTPA crystals.

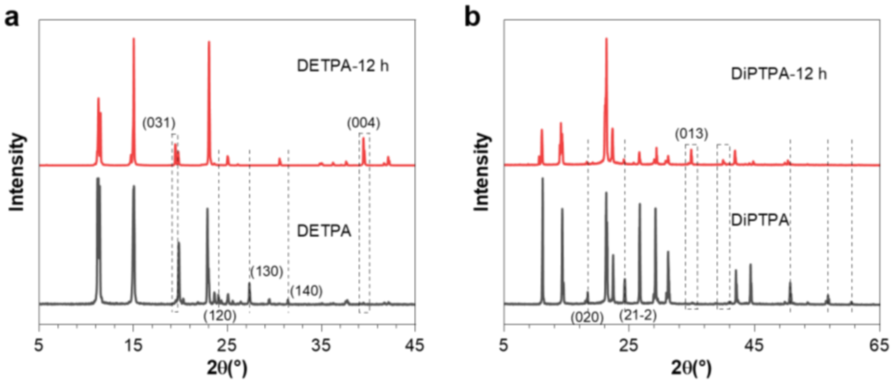

Supplementary Fig. 45. XRD characterization of DETPA and DiPTPA crystals. XRD patterns of (a) DETPA and (b) DiPTPA crystals before and after 12 h of 312 nm UV irradiation.

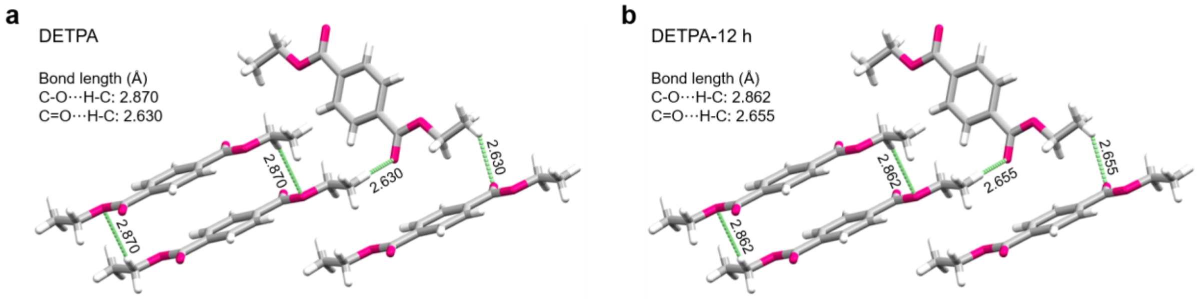

Supplementary Fig. 46. Evolution of bond lengths in DETPA crystals. The transformation of bond lengths of denoted intermolecular interactions in DETPA crystal (a) before and (b) after 12 h of 312 nm UV irradiation.

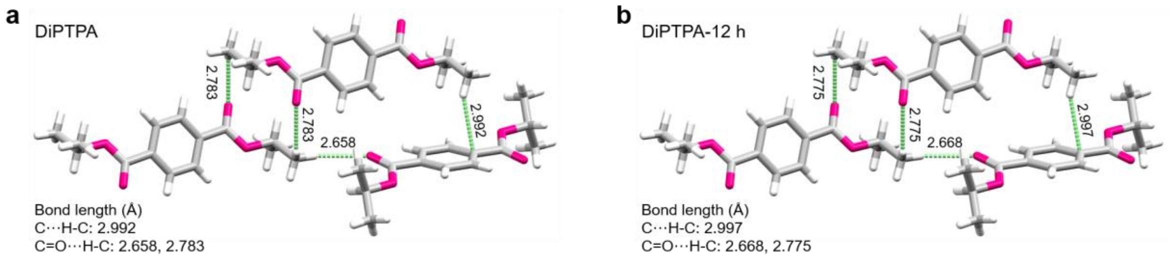

Supplementary Fig. 47. Evolution of bond lengths in DiPTPA crystals. The transformation of bond lengths of denoted intermolecular interactions in DiPTPA crystal (a) before and (b) after 12 h of 312 nm UV irradiation.

Supplementary Table 2. Single crystal data of DETPA before and after 12 h of 312 nm UV irradiation.

	DETPA	DETPA-12 h
Formula	$C_{12}H_{14}O_4$	$C_{12}H_{14}O_4$
Formula Weight	222.23	222.23
Wavelength (Å)	1.54178	1.54178
Space Group	P21/n	P21/n
Cell Length (Å)	a=4.2243 (5)<br>b=15.4519 (15)<br>c=9.1426 (11)	a=4.2253 (7)<br>b=15.441 (3)<br>c=9.1422 (14)
Cell Angle (°)	α=90<br>β=92.902 (8)<br>γ=90	α=90<br>β=92.968 (10)<br>γ=90
Cell Volume (Å^3)	596.00 (12)	595.68 (17)
Z	2	2
Density (g/cm^3)	1.238	1.239
F (000)	236.0	236.0
h_{max}, k_{max}, l_{max}	5, 18, 10	5, 18, 11
T_{min}, T_{max}	0.597, 0.753	0.533, 0.753

Supplementary Table 3. Single crystal data of DiPTPA before and after 12 h of 312 nm UV irradiation.

	DiPTPA	DiPTPA-12 h
Formula	$C_{14}H_{18}O_4$	$C_{14}H_{18}O_4$
Formula Weight	250.28	250.28
Wavelength (Å)	1.54178	1.54178
Space Group	P21/c	P21/n
Cell Length (Å)	a= 9.0928 (8)<br>b= 9.6861 (9)<br>c= 9.2091 (10)	a= 9.1032 (6)<br>b= 9.6809 (6)<br>c= 9.2082 (7)
Cell Angle (°)	α=90<br>β=119.578 (4)<br>γ=90	α=90<br>β=119.607 (3)<br>γ=90
Cell Volume (Å^3)	705.38 (12)	705.54 (9)
Z	2	2
Density (g/cm^3)	1.178	1.178
F (000)	268.0	268.0
h_{max}, k_{max}, l_{max}	10, 11, 10	10, 11, 11
T_{min}, T_{max}	0.609, 0.753	0.632, 0.753

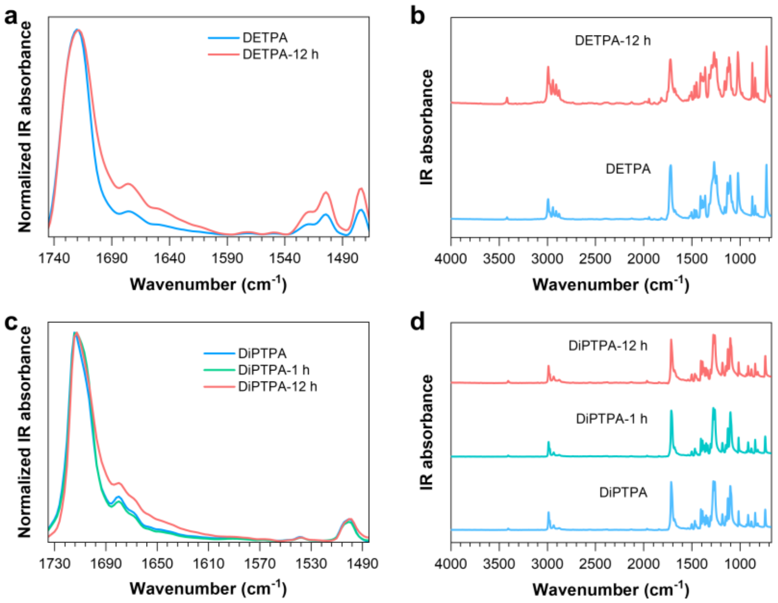

Supplementary Fig. 48. FTIR characterization of DETPA and DiPTPA crystals. (a,c) The local and (b,d) the whole range of the FTIR spectra of (a,b) DETPA and (c,d) DiPTPA crystals with different exposure time under 312 nm UV irradiation.

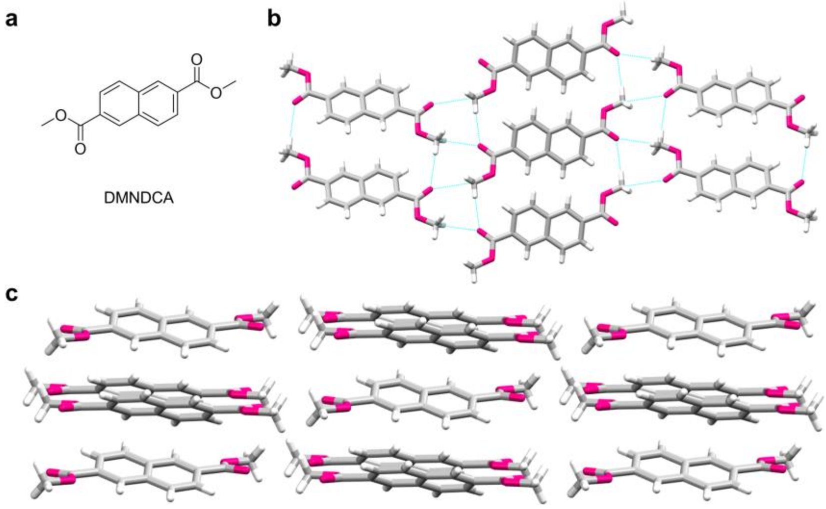

Supplementary Fig. 49. Single crystal analysis of DMNDCA. (a) The structural formula of DMNDCA. (b, c) Single crystal structure and fragmental molecular packing of DMNDCA.

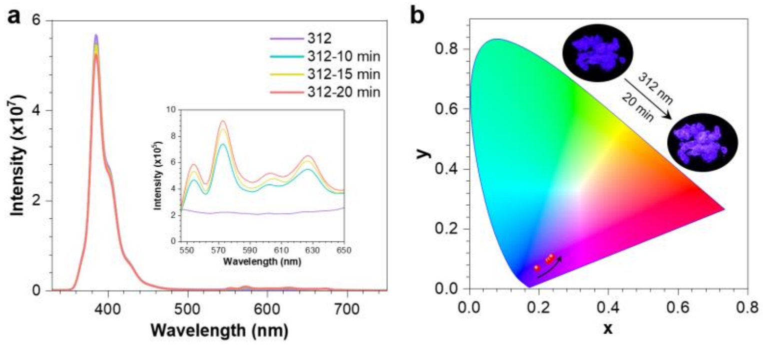

Supplementary Fig. 50. PCL process of DMNDCA crystals. (a) Prompt emission spectra, (b) CIE coordinate diagram and (b, inset) luminescent photographs of PL colour transforming process of DMNDCA crystals under 312 nm UV irradiation with different exposure times. The inset in (a) is a larger version of the spectra from 545 to 650 nm. The CIE coordinates and colour transformation trend are labelled by red dots and arrow, respectively.

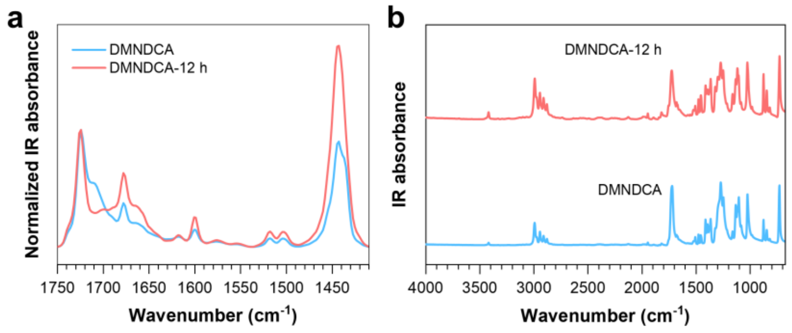

Supplementary Fig. 51. FTIR characterization of DMNDCA crystals. (a) The local and (b) the whole range of the FTIR spectra of DMNDCA crystals before and after 12 h of 312 nm UV irradiation.

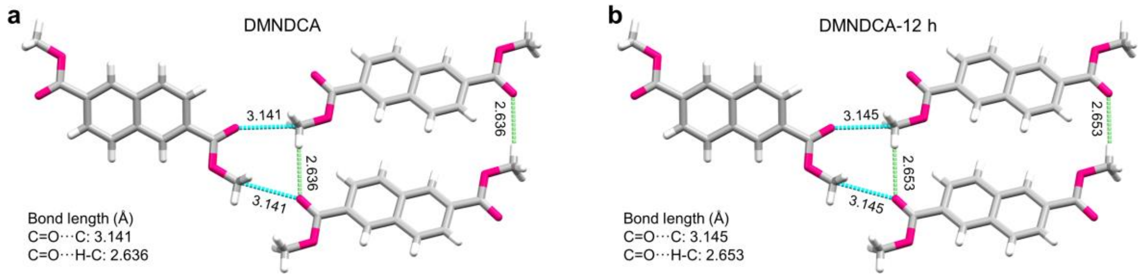

Supplementary Fig. 52. Evolution of bond lengths in DMNDCA crystals. The transformation of bond lengths of denoted intermolecular interactions in DMNDCA crystal (a) before and (b) after 12 h of 312 nm UV irradiation.

Supplementary Table 4. Single crystal data of DMNDCA before and after 12 h of 312 nm UV irradiation.

	DMNDCA	DMNDCA-12 h
Formula	$C_{14}H_{12}O_4$	$C_{14}H_{12}O_4$
Formula Weight	244.24	244.24
Wavelength (Å)	1.54178	1.54178
Space Group	P21/c	P21/n
Cell Length (Å)	a= 13.4344 (7)<br>b= 6.1512 (3)<br>c= 7.1638 (4)	a= 13.4478 (7)<br>b= 6.1584 (3)<br>c= 7.1669 (4)
Cell Angle (°)	α=90<br>β= 100.415 (2)<br>γ=90	α=90<br>β= 100.366 (4)<br>γ=90
Cell Volume (Å^3)	582.25 (5)	583.85 (5)
Z	2	2
Density (g/cm^3)	1.393	1.389
F (000)	256.0	256.0
h_{max}, k_{max}, l_{max}	16, 7, 8	16, 7, 8
T_{min}, T_{max}	0.596, 0.753	0.603, 0.753

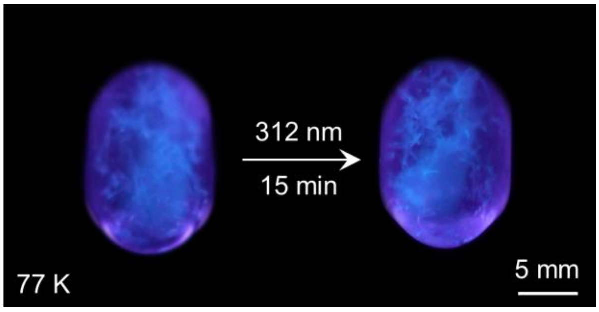

Supplementary Fig. 53. PCL process is inhibited at 77 K. The luminescent photographs of DMTPA crystals at 77 K before and after 15 min of 312 nm UV irradiation. The PCL phenomenon is impeded at such cryogenic circumstance.

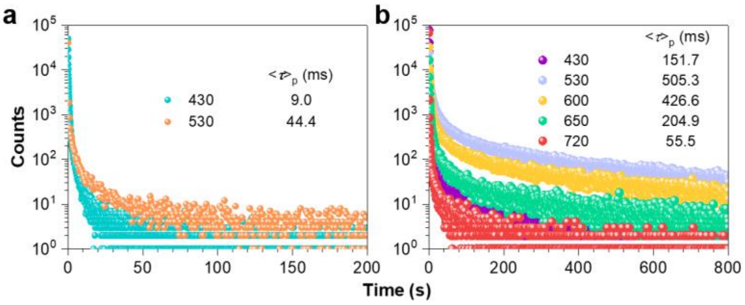

Supplementary Fig. 54. Lifetime profiles of MMTPA crystals. Millisecond-scale lifetimes for MMTPA crystals (a) before and (b) after 312 nm UV irradiation. (λ_{ex} = 312 nm).

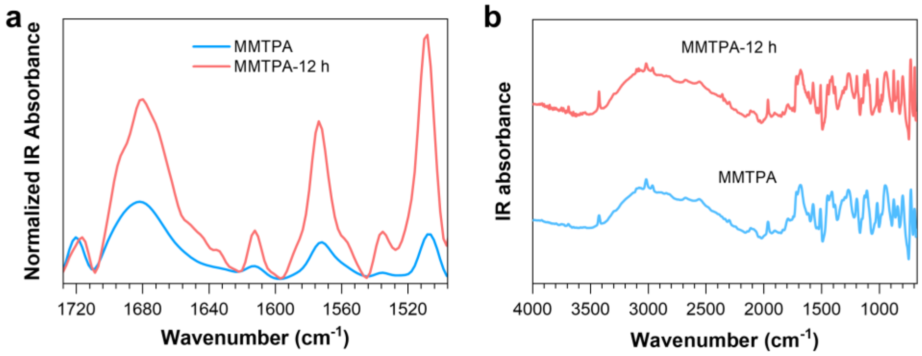

Supplementary Fig. 55. FTIR characterization of MMTPA crystals. (a) The local and (b) the whole range of the FTIR spectra of MMTPA crystals before and after 12 h of 312 nm UV irradiation.

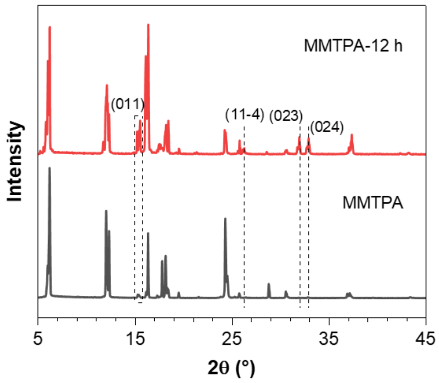

Supplementary Fig. 56. XRD characterization of MMTPA crystals. XRD patterns of MMTPA crystals before and after 12 h of 312 nm UV irradiation.

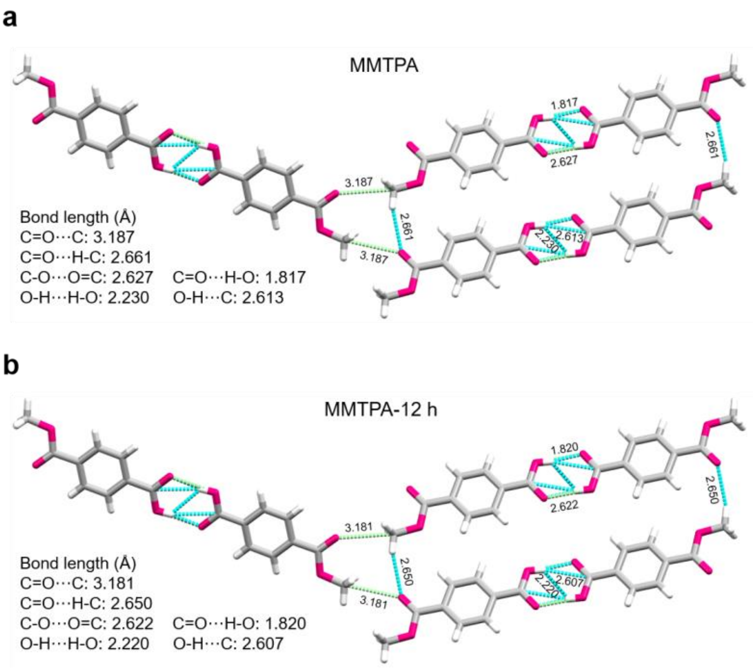

Supplementary Fig. 57. Evolution of bond lengths in MMTPA crystals. The transformation of bond lengths of denoted intermolecular interactions in MMTPA crystal (a) before and (b) after 12 h of 312 nm UV irradiation.

Supplementary Table 5. Single crystal data of MMTPA before and after 12 h of 312 nm UV irradiation.

	MMTPA	MMTPA-12 h
Formula	$C_9H_8O_4$	$C_9H_8O_4$
Formula Weight	180.15	180.15
Wavelength (Å)	1.54178	1.54178
Space Group	P21/c	P21/n
Cell Length (Å)	a= 4.8531 (11)<br>b= 5.8842 (11)<br>c= 29.172 (6)	a= 4.840 (2)<br>b= 5.8675 (18)<br>c=29.145 (8)
Cell Angle (°)	α=90<br>β= 91.557 (10)<br>γ=90	α=90<br>β= 91.52 (2)<br>γ=90
Cell Volume (Å^3)	832.8 (3)	827.4 (5)
Z	4	4
Density (g/cm^3)	1.437	1.446
F (000)	376.0	376.0
h_{max}, k_{max}, l_{max}	5, 7, 35	5, 7, 35
T_{min}, T_{max}	0.715, 0.753	0.566, 0.753

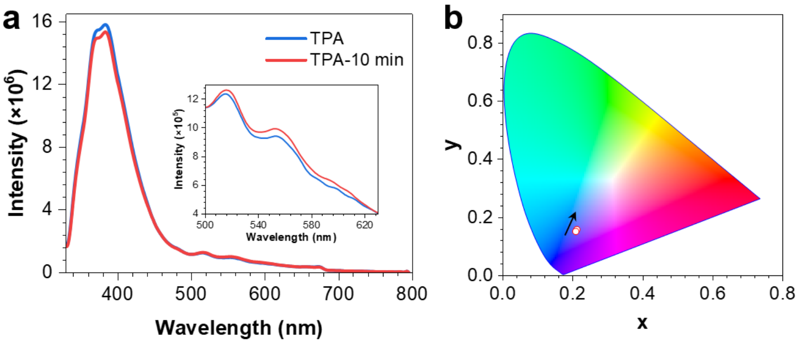

Supplementary Fig. 58. Nearly no PCL process is found in TPA powder crystals. (a) Prompt emission spectra and (b) CIE coordinate diagram of PL colour transforming process of TPA powder crystals before and after 10 min of 312 nm UV irradiation. The inset in (a) is a larger version of the spectra from 500 to 630 nm. The CIE coordinates and colour transformation trend are labelled by white dots and arrow, respectively.

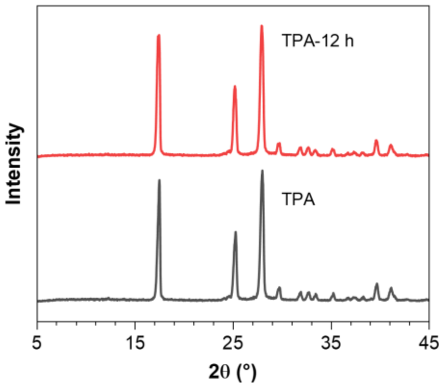

Supplementary Fig. 59. XRD characterization of TPA powder crystals. XRD patterns of TPA powder crystals before and after 12 h of 312 nm UV irradiation.

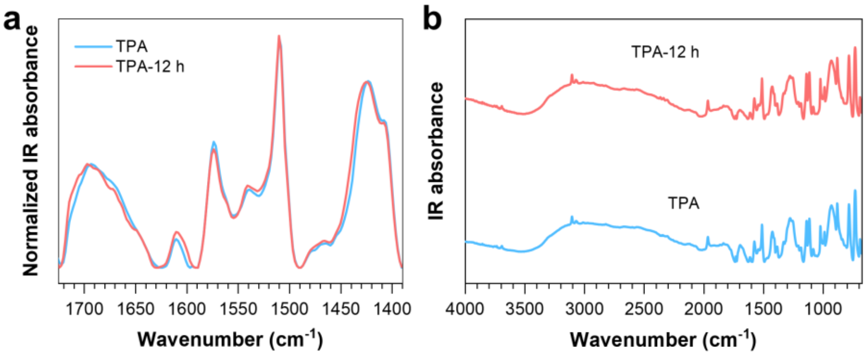

Supplementary Fig. 60. FTIR characterization of TPA powder crystals. (a) The local and (b) the whole range of the FTIR spectra of TPA powder crystals before and after 12 h of 312 nm UV irradiation.

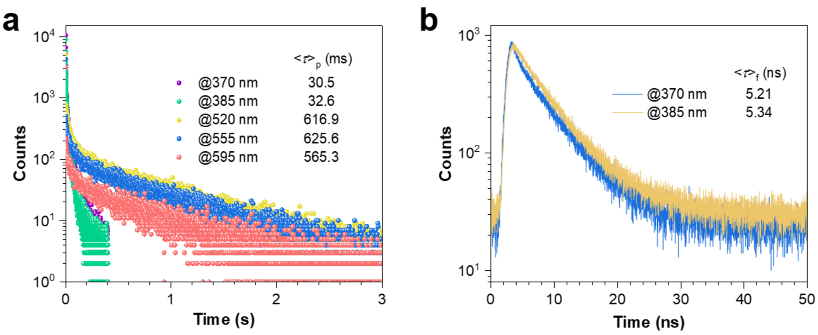

Supplementary Fig. 61. Lifetime profiles of TPA powder crystals. (a) Millisecond- and (b) nanosecond-scale lifetimes for TPA powder crystals (λ_{ex} = 312 nm).

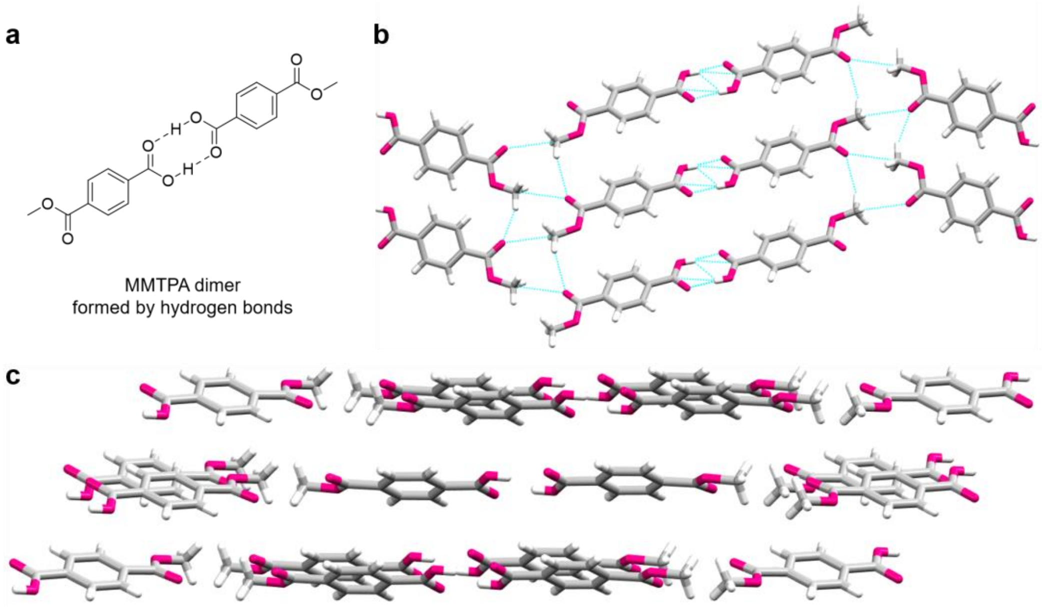

Supplementary Fig. 62. Single crystal analysis of MMTPA. (a) The structural formula of MMTPA dimer connected by hydrogen bonds. (b, c) Single crystal structure and fragmental molecular packing of MMTPA.

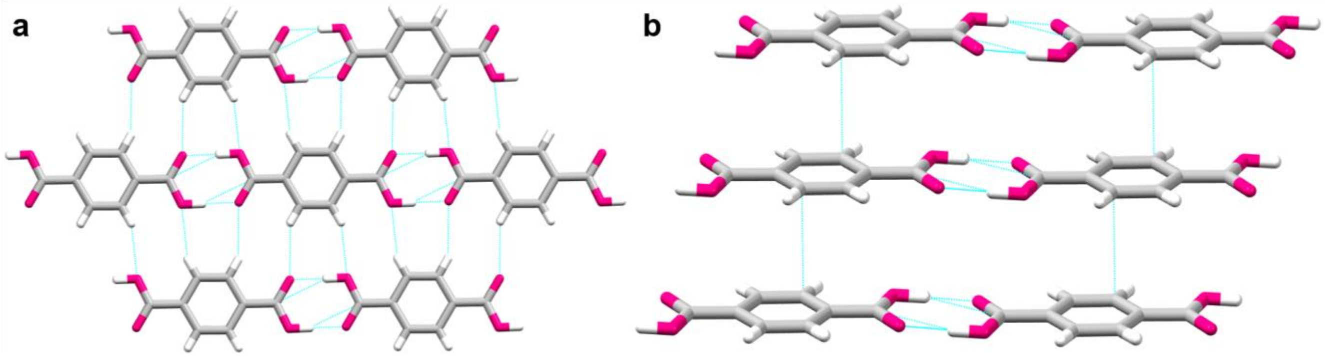

Supplementary Fig. 63. Single crystal analysis of TPA. Single crystal structure and fragmental (a) intra- and (b) interlayer molecular packing of TPA[3].

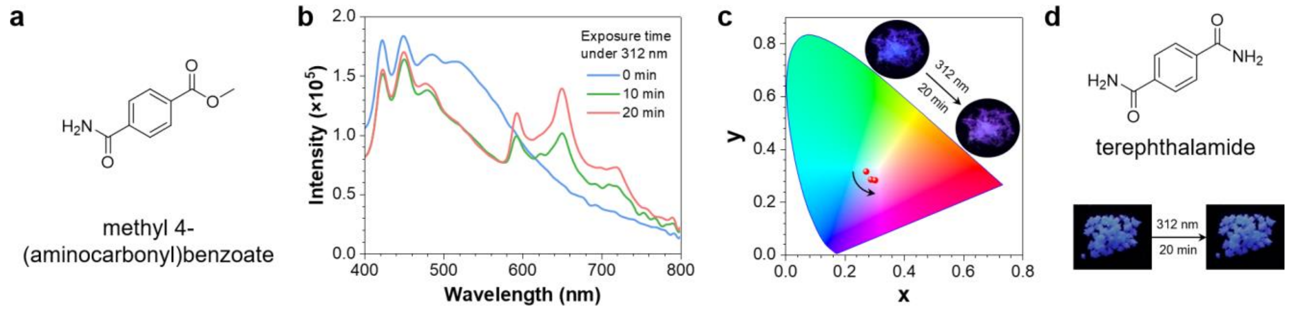

Supplementary Fig. 64. PCL process of methyl 4-(aminocarbonyl)benzoate crystals. (a) The structural formula of methyl 4-(aminocarbonyl)benzoate. (b) Prompt emission spectra, (c) CIE coordinate diagram and (c, inset) luminescent photographs of PL colour transforming process of methyl 4-(aminocarbonyl)benzoate crystals under 312 nm UV irradiation with different exposure times (λ_{ex} = 312 nm). (d) The structural formula and luminescent photographs of terephthalamide. The PL colour of its crystals seldom transforms after 20 min of 312 nm UV irradiation. The CIE coordinates and colour transformation trend are labelled by red dots and arrow, respectively.

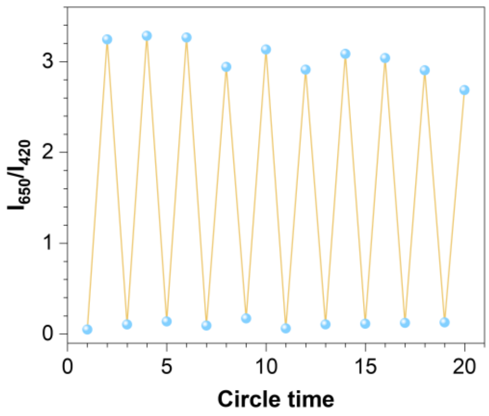

Supplementary Fig. 65. Cyclic stability of the PL transformation process of DMTPA/PAM hydrogel. Plot of luminescence intensity ratio versus repeated phototriggering and heating cycles. I_{650} and I_{420} refer to the PL intensities at 650 and 420 nm, respectively (λ_{ex} = 312 nm).

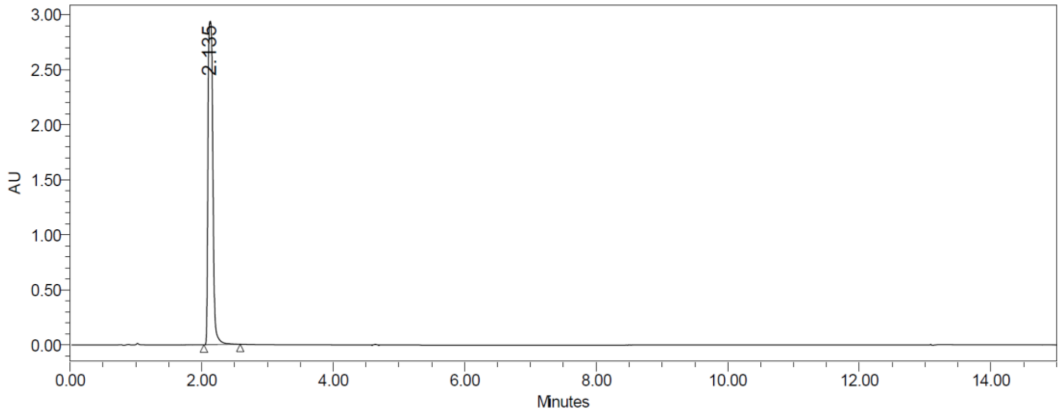

Supplementary Fig. 66. HPLC analysis for MMTPA. HPLC results for MMTPA crystals. The y-axis unit AU represents absorbance unit.

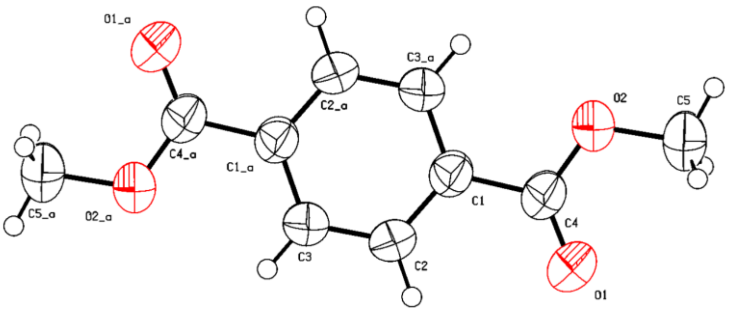

Supplementary Fig. 67. ORTEP-style illustration of DMTPA (CCDC number 2299585) with probability ellipsoids.

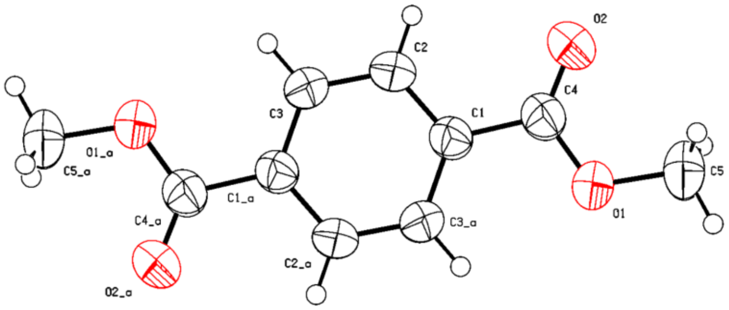

Supplementary Fig. 68. ORTEP-style illustration of DMTPA-5 min (CCDC number 2299586) with probability ellipsoids.

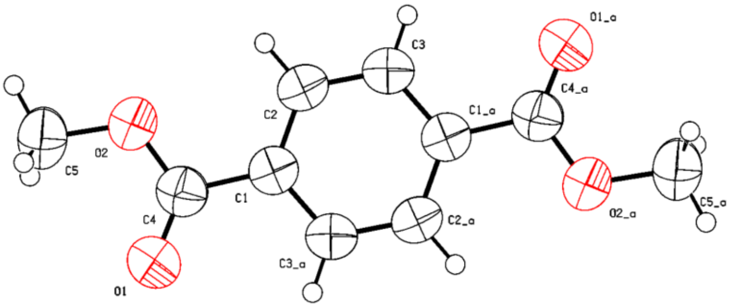

Supplementary Fig. 69. ORTEP-style illustration of DMTPA-12 h (CCDC number 2299587) with probability ellipsoids.

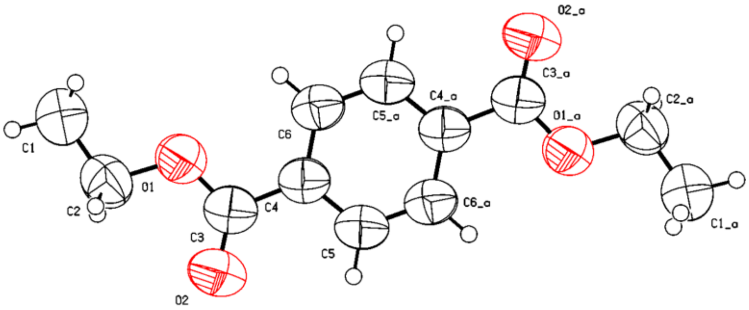

Supplementary Fig. 70. ORTEP-style illustration of DETPA (CCDC number 2299619) with probability ellipsoids.

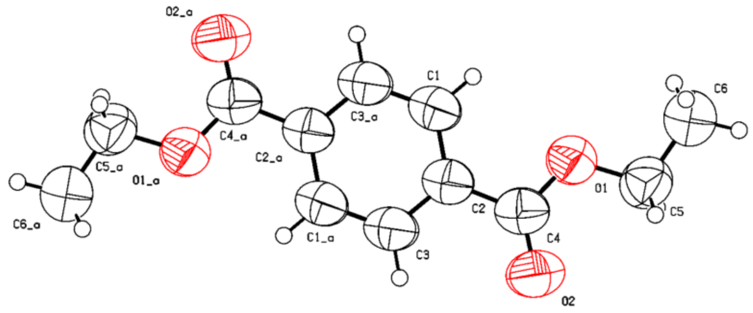

Supplementary Fig. 71. ORTEP-style illustration of DETPA-12 h (CCDC number 2299620) with probability ellipsoids.

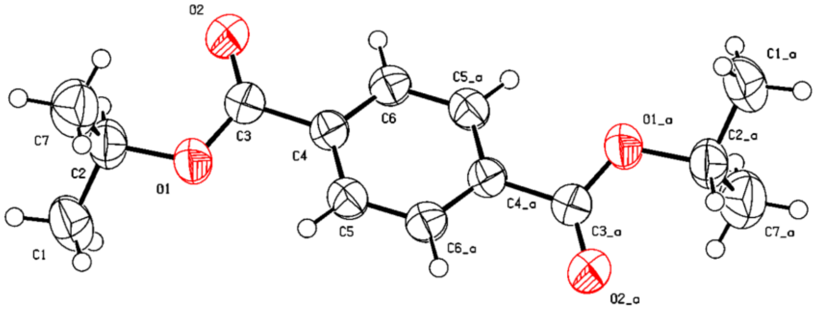

Supplementary Fig. 72. ORTEP-style illustration of DiPTPA (CCDC number 2299594) with probability ellipsoids.

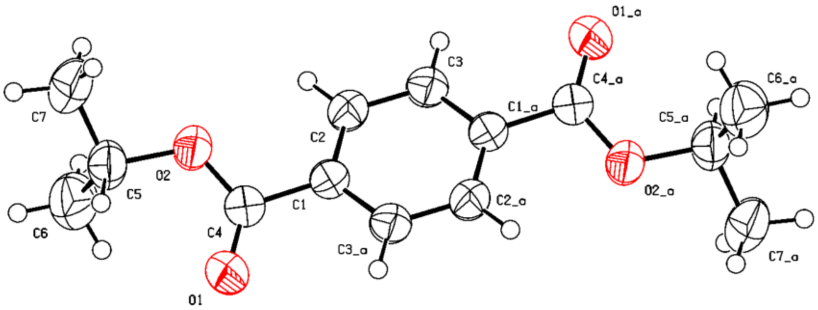

Supplementary Fig. 73. ORTEP-style illustration of DiPTPA-12 h (CCDC number 2299595) with probability ellipsoids.

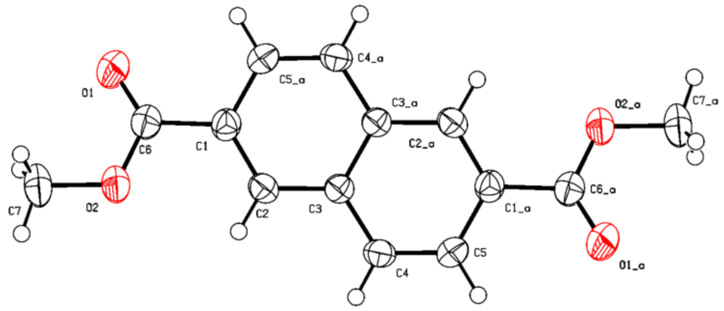

Supplementary Fig. 74. ORTEP-style illustration of DMNDCA (CCDC number 2299598) with probability ellipsoids.

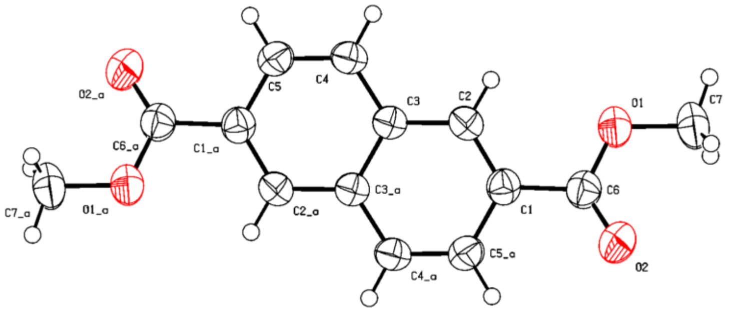

Supplementary Fig. 75. ORTEP-style illustration of DMNDCA-12 h (CCDC number 2299599) with probability ellipsoids.

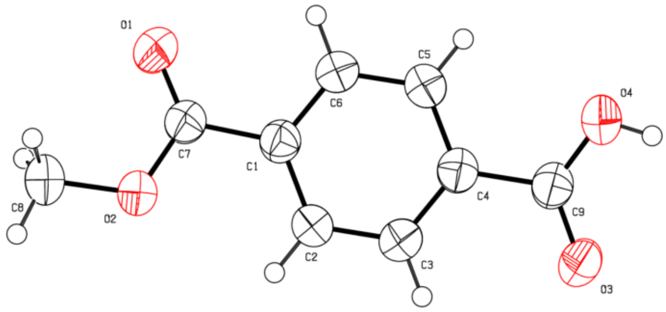

Supplementary Fig. 76. ORTEP-style illustration of MMTPA (CCDC number 2299600) with probability ellipsoids.

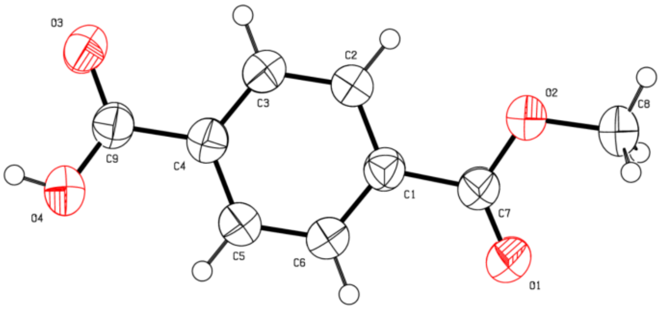

Supplementary Fig. 77. ORTEP-style illustration of MMTPA-12 h (CCDC number 2299601) with probability ellipsoids.

Supplementary References

1. Yang, G.-W., Zhang, Y.-Y. Xie, R. & Wu, G.-P. Scalable bifunctional organoboron catalysts for copolymerization of CO_2 and epoxides with unprecedented efficiency. *J. Am. Chem. Soc.* **142**, 12245 (2020).
2. Johnson, E. R., Keinan, S., Mori-Sánchez, P., Contreras-García, J., Cohen, A. J. & Yang, W. Revealing noncovalent interactions. *J. Am. Chem. Soc.* **132**, 6498 (2010).
3. Ahmed, E., Karothu, D. P., Warren, M. & Naumov, P. Shape-memory effects in molecular crystals. *Nat. Commun.* **10**, 3723 (2019).
