# Supplementary material for: Photochromic luminescence of organic crystals arising from subtle molecular rearrangement
Source: Nat Commun. 2024 Jun 13;15:5054. doi: 10.1038/s41467-024-48728-w (PMC11176386; doi:10.1038/s41467-024-48728-w)
Supplement: Supplementary file 3 — Description of Additional Supplementary Files [file 41467_2024_48728_MOESM3_ESM.pdf]

Description of additional supplementary files:

Supplementary Movie 1: The photochromic luminescent process of DMTPA crystals under 312 nm UV irradiation. The quantity of samples, ~30 mg; power density: 3.5 mW/cm^2. The movie speed is labelled in the bottom right corner of the video.
